# Combined Nurr1 and Foxa2 roles in the therapy of Parkinson's disease

**DOI:** 10.15252/emmm.201404610

**Published:** 2015-03-10

**Authors:** Sang-Min Oh, Mi-Yoon Chang, Jae-Jin Song, Yong-Hee Rhee, Eun-Hye Joe, Hyun-Seob Lee, Sang-Hoon Yi, Sang-Hun Lee

**Affiliations:** 1 Department of Biochemistry and Molecular Biology, College of Medicine, Hanyang UniversitySeoul, Korea; 2 Hanyang Biomedical Research Institute, Hanyang UniversitySeoul, Korea; 3 Graduate School of Biomedical Science and Engineering, Hanyang UniversitySeoul, Korea; 4 Department of Phamacology, Ajou University School of MedicineSuwon, Korea; 5 Department of Applied Bioscience, College of Life Science, CHA UniversitySeoul, Korea

**Keywords:** Foxa2, gene therapy, midbrain dopamine neuron, Nurr1, Parkinson's disease

## Abstract

Use of the physiological mechanisms promoting midbrain DA (mDA) neuron survival seems an appropriate option for developing treatments for Parkinson's disease (PD). mDA neurons are specifically marked by expression of the transcription factors Nurr1 and Foxa2. We show herein that Nurr1 and Foxa2 interact to protect mDA neurons against various toxic insults, but their expression is lost during aging and degenerative processes. In addition to their proposed cell-autonomous actions in mDA neurons, forced expression of these factors in neighboring glia synergistically protects degenerating mDA neurons in a paracrine mode. As a consequence of these bimodal actions, adeno-associated virus (AAV)-mediated gene delivery of Nurr1 and Foxa2 in a PD mouse model markedly protected mDA neurons and motor behaviors associated with nigrostriatal DA neurotransmission. The effects of the combined gene delivery were dramatic, highly reproducible, and sustained for at least 1 year, suggesting that expression of these factors is a promising approach in PD therapy.

## Introduction

Parkinson's disease (PD) is a common movement disorder prevalent in older persons. The pathophysiologic feature of PD is progressive degeneration of dopamine (DA)-secreting neurons (DA neurons) in the substantia nigra (SN) of the midbrain, which results in loss of nigrostriatal DA neurotransmission. Midbrain DA (mDA) neurons in the SN are particularly vulnerable to oxidative stress because of their reduced levels of the antioxidant glutathione and increased nigral iron content (Dexter *et al*, 1989; Sian *et al*, 1994). An estimated 5–10% of all PD cases are associated with genetic mutations of the cellular components that scavenge reactive oxygen species (ROS) (Dias *et al*, 2013). In addition, chronic inflammation underlies PD pathology, and a number of PD risk factors such as environmental toxins, heavy metals, head trauma, and bacterial or viral infections are underpinned by inflammation (Wirdefeldt *et al*, 2011). Inflammatory reactions induce the production of ROS, and the reverse also occurs. Thus a feed-forward cycle of ROS production and inflammation underlies DA neuronal cell death in idiopathic PD.

Current PD therapies predominantly aim at restoring DA levels or correcting functional perturbation of the basal ganglia caused by DA loss. These symptomatic therapies, however, do not slow down disease progression and their efficacy declines over time. The search continues for a treatment strategy that targets intrinsic pathways promoting the viability and resistance of mDA neurons against toxic insults. In addition, the idea of modifying the disease environment surrounding mDA neurons is appearing on the therapeutic horizon. Glial cells can produce both detrimental and beneficial environments in the central nervous system (CNS), and these are associated with the release of pro-inflammatory and anti-inflammatory (or neurotrophic) cytokines, respectively (reviewed in reference Neumann *et al*, 2009). Thus a strategy biasing toward beneficial glial cells could be a promising therapeutic approach in PD.

Nuclear receptor-related factor 1 (Nurr1, also known as NR4A2) is an orphan nuclear receptor initially characterized as a transcription factor important for mDA neuron development, comprising the generation (Zetterstrom *et al*, 1997), maturation (Castro *et al*, 2001), and axonal pathfinding of mDA neurons (Wallen *et al*, 1999). Nurr1 continues to be expressed in adult mDA neurons, and adult-onset deletion of this protein leads to progressive loss of mDA neurons (Kadkhodaei *et al*, 2009). In heterozygous Nurr1 mice, mDA neurons are more vulnerable to dopaminergic neurotoxins (Le *et al*, 1999). The Nurr1 level in mDA neurons decreases in the elderly (Chu *et al*, 2002) and PD patients (Bauer *et al*, 2008), and polymorphisms and mutations resulting in reduced expression of Nurr1 are associated with familial and sporadic PD (Le *et al*, 2003; Zheng *et al*, 2003; Hering *et al*, 2004). These findings support the notion that Nurr1 exerts a protective effect on adult mDA neurons in a cell-autonomous manner. Indeed, several intrinsic mechanisms implicated in Nurr1-mediated cell survival have been identified (Volakakis *et al*, 2010; Malewicz *et al*, 2011; Decressac *et al*, 2012; Kadkhodaei *et al*, 2013). In addition to the intrinsic role of Nurr1 in mDA neurons, a recent study (Saijo *et al*, 2009) has identified an unexpected Nurr1 effect in which it is expressed in glial cells in response to stimuli triggering inflammation, and this Nurr1 suppresses the production of inflammatory mediators that cause the death of mDA neurons. These findings collectively indicate that Nurr1 could be a promising therapeutic target (Decressac *et al*, 2013b), by which degenerating mDA neurons could be protected in a bidirectional therapeutic mode by promoting intracellular survival pathways in mDA neurons as well as by modulating toxic environments surrounding these neurons.

Nurr1 belongs to the family of steroid nuclear hormone receptors whose activities are largely regulated by co-regulators in the form of repressor (NCoR) and activator (NCoA) complexes (Xu *et al*, 1999). This suggests that a sufficient therapeutic effect, if any effect occurred at all, might not be achieved with Nurr1 alone in the absence of co-activators. We and others have recently shown that Foxa2 of the forkhead family of winged-helix transcription factors (also known as hepatocyte nuclear factor 3beta, HNF3β) is a potent co-activator of the effect of Nurr1 on the production of mDA neurons (Lee *et al*, 2010; Yi *et al*, 2014) and their maintenance (Stott *et al*, 2013) during midbrain development. It is noteworthy that expression of Foxa2, like Nurr1, continues in adult mDA neurons after the termination of development. As in Nurr1-knockout mice, DA neuronal death is evident in the midbrains of Foxa2 haplo-insufficiency mice (Kittappa *et al*, 2007). These findings, collectively, suggest that Nurr1 and Foxa2 act together in the adult midbrain to protect mDA neurons. In this study, we evaluated the therapeutic potential of co-expressing Nurr1 and Foxa2 in the degenerating midbrains of a mouse PD model.

## Results

### Nurr1 and Foxa2 interact to protect mDA neurons against toxic insult

**Figure 1:**
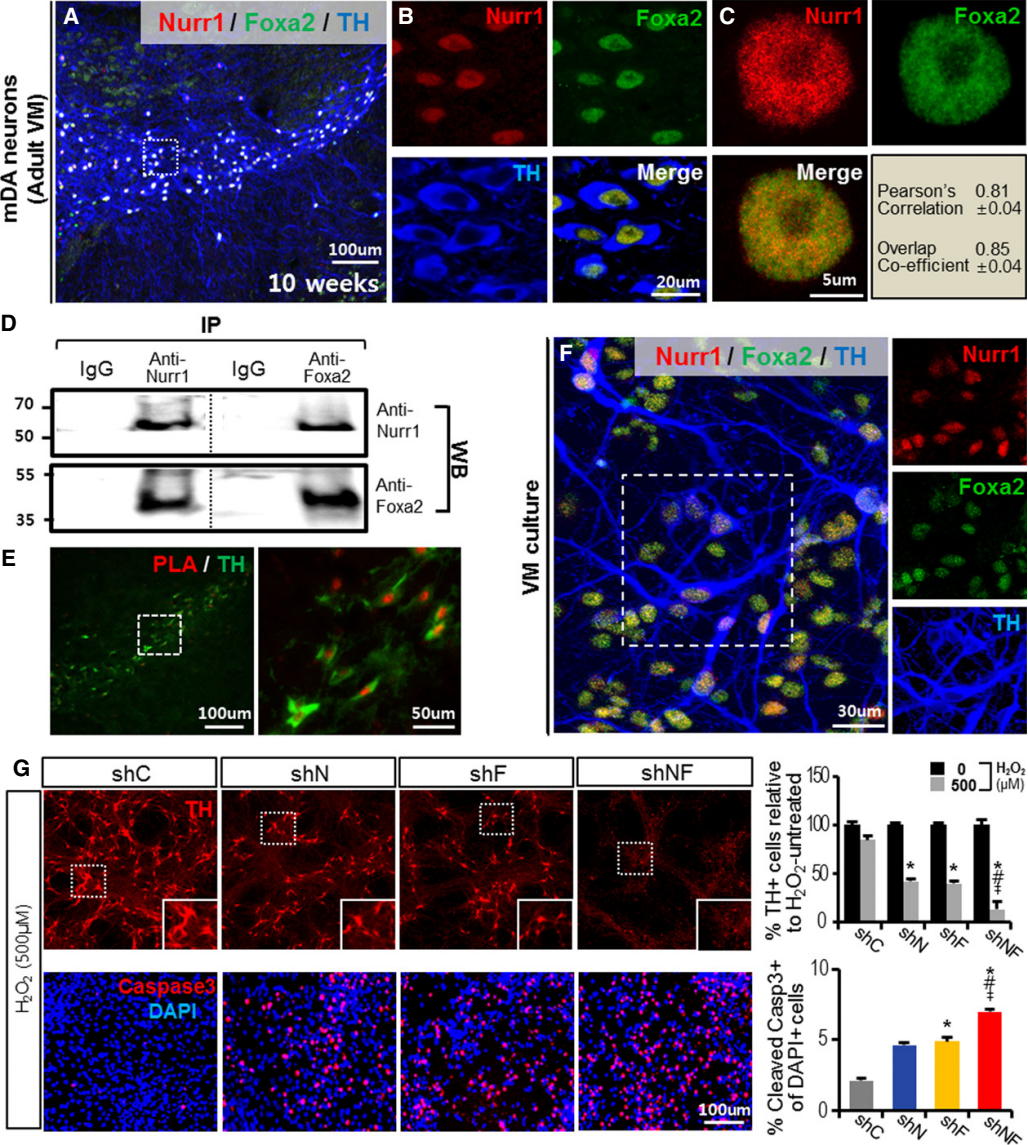
Nurr1 and Foxa2 interact physically and functionally to protect mDA neurons from toxic insult

Confocal microscopic analysis on the midbrains of adult mice (10 weeks old) demonstrated that Nurr1 and Foxa2 were present in the nuclei of virtually all tyrosine hydroxylase (TH)-positive mDA neurons (Fig1A and B) with high colocalization indices (Fig1C). In immunoprecipitation (IP) experiments using midbrain lysates of 10-week-old mice, Foxa2 was detected in Nurr1 antibody precipitates and Nurr1 was also detected in Foxa2 antibody precipitates (Fig1D), demonstrating physical binding between Nurr1 and Foxa2 in the adult midbrain. Close physical interaction of these two proteins was confirmed by an *in situ* proximity ligation assay (PLA), which allows visualization of protein–protein binding by the red fluorescence (554 nm) emanating from two proteins in close proximity (Fig1E). These findings collectively suggest an interplay between Nurr1 and Foxa2 that promotes mDA neuron functions in the adult midbrain, like those seen in the developing midbrain (Lee *et al*, 2010; Stott *et al*, 2013; Yi *et al*, 2014).

A–C Co-localization of Nurr1 and Foxa2 proteins in mDA neurons of the adult mouse midbrain. Representative confocal sections of the ventral midbrain (A) stained with antibodies specific for TH, Nurr1, and Foxa2. The midbrains of mice at 10 weeks were cryosectioned, and stained images were taken using a confocal microscope with *z*-stacks through the section thickness (12 μm). Shown in (B) are individual and merged images of TH, Nurr1, and Foxa2 staining of the boxed area in (A) at higher magnification. Representative images of a single nucleus from a TH^+^ mDA neuron of an adult midbrain section (C) co-stained with Nurr1 (red) and Foxa2 (green). Co-localization of Nurr1 and Foxa2 in the merged images was assessed by Pearson's correlation and overlap coefficient values (*n* = 4, right/low quadrant in C).

D Immunoprecipitation (IP) assay for Nurr1 and Foxa2 protein binding. The ventral part of midbrains was dissected from 10-week-old mice, lysed, and subjected to IP. Nurr1/Foxa2 protein binding was detected by WB analysis using an anti-Foxa2 antibody in immunoprecipitates generated with anti-Nurr1 antibody (left), as well as by anti-Nurr1 WB assay in immunoprecipitates with anti-Foxa2 antibody (right).

E Physical interaction between Nurr1 and Foxa2 was further assessed by a proximity ligation assay (PLA). The SN area of a midbrain section (10 weeks old) was subjected to the PLA reaction and counterstained for TH. The boxed area in the left panel exhibiting physical Nurr1/Foxa2 interaction (red) in TH^+^ DA neurons (green) is enlarged in the right panel.

F, G Knockdown of Nurr1 and Foxa2 synergistically aggravates H_2_O_2_-induced cell death of mDA neurons in culture. Representative image for Nurr1- and Foxa2-co-expressing mDA neurons (F) used in the loss-of-function study. Shown in right panels are the individual Nurr1-, Foxa2-, and TH-stained cells of the boxed area. Nurr1- and Foxa2-co-expressing mDA neurons were formed after 9 days of differentiation in VM-NPC cultures *in vitro* and transduced with lentiviruses expressing shNurr1 (shN), shFoxa2 (shF), shN + shF (shNF), or shControl (shC). Three days later, the cultures were treated with H_2_O_2_ (500 μM) for 8 h and cells positive for TH (upper) and cleaved (activated) caspase-3 (lower) were counted the following day (G). Insets, high-power TH^+^ cell images of the boxed areas. Significantly different from the control (shC)*, shN^#^, shF^‡^ at *P* < 0.05, *n* = 5 culture wells in each group. *P*-values: 0.038 (shN*), 0.026 (shF*), 0.013 (shNF*), 0.036 (shNF^#^), and 0.042 (shNF^‡^) for the % TH^+^ cells; 0.043 (shF*), 0.037 (shNF*), 0.019 (shNF^#^), and 0.029 (shNF^‡^) for the percent cleaved caspase-3-positive cells; one-way ANOVA followed by Bonferroni *post hoc* test.

Midbrain-type DA neurons co-expressing endogenous Nurr1 and Foxa2 were generated by *in vitro* differentiation of neural precursor cells (NPCs) derived from the ventral midbrain (VM) (Fig1F, reference Yi *et al*, 2014); the characteristics of these cultures are described further in Supplementary Fig S1. To examine the roles of Nurr1 and Foxa2, differentiated cultures were treated with silencing small hairpin RNAs (shRNAs) for these factors. The shRNA treatments were effective in down-regulating Nurr1 and Foxa2 expression. mRNA levels of Nurr1 (relative to the shControl) were l ± 0.03, 0.43 ± 0.01, 0.84 ± 0.03, and 0.28 ± 0.004, in the presence of shControl, shNurr1, shFoxa2, and shNurr1 + shFoxa2, respectively, and the corresponding Foxa2 mRNA levels were 1 ± 0.05, 0.89 ± 0.03, 0.45 ± 0.06, and 0.33 ± 0.02 (*n* = 3 PCRs for each group). These results are consistent with a positive regulatory loop for Nurr1 and Foxa2 expression (Yi *et al*, 2014), since Nurr1 expression was slightly decreased by shFoxa2 treatment, and Foxa2 expression was slightly decreased by shNurr1 treatment. Most importantly, DA neuronal numbers were synergistically decreased by Nurr1 and Foxa2 knockdown: TH^+^ cells at 3 days after the shRNA treatments were 1,235 ± 17 (shControl), 816 ± 13 (shNurr1), 805 ± 14 (shFoxa2), and 276 ± 7 cells/well (shNurr1 + shFoxa2) (*n* = 4 culture wells in each group), indicating that Nurr1 and Foxa2 cooperate in promoting mDA neuronal survival. Treatment with the free radical-producing chemical H_2_O_2_ caused mDA neuronal death. The loss of TH^+^ mDA neurons in response to H_2_O_2_ was increased in cultures treated with shNurr1 or shFoxa2, and this was accompanied by increased numbers of cells with cleaved (activated) caspase-3 (Fig1G). The effect of combined shNurr1 + shFoxa2 treatment was even more dramatic. Indeed, although a few cells remained immunoreactive for TH in the cultures treated with shNurr1 + shFoxa2, all had blunted or fragmented neurites (Fig1G inset), a neuronal aging and degenerative phenotype (Hof & Morrison, 2004). Because of the developmental roles of Nurr1 and Foxa2 in mDA neuron generation (Yi *et al*, 2014), the observed effects of shNurr1 + shFoxa2 might have been obtained by affecting mDA neurogenesis. However, we did not detect any new neuron formation in the differentiated cultures (Supplementary Fig S1A–C), and shNurr1 and shFoxa2 treatment did not alter neurogenesis in BrdU-pulse experiments (Supplementary Fig S1C). Collectively, these findings suggest that Nurr1 and Foxa2 interact physically and functionally in mDA neurons to protect these neurons from toxic insults, and their neuroprotective effects are probably mediated in a cell-autonomous manner since these factors are actually present in the neurons.

### Nurr1 and Foxa2 protein levels decrease in mDA neurons during aging and degeneration

**Figure 2:**
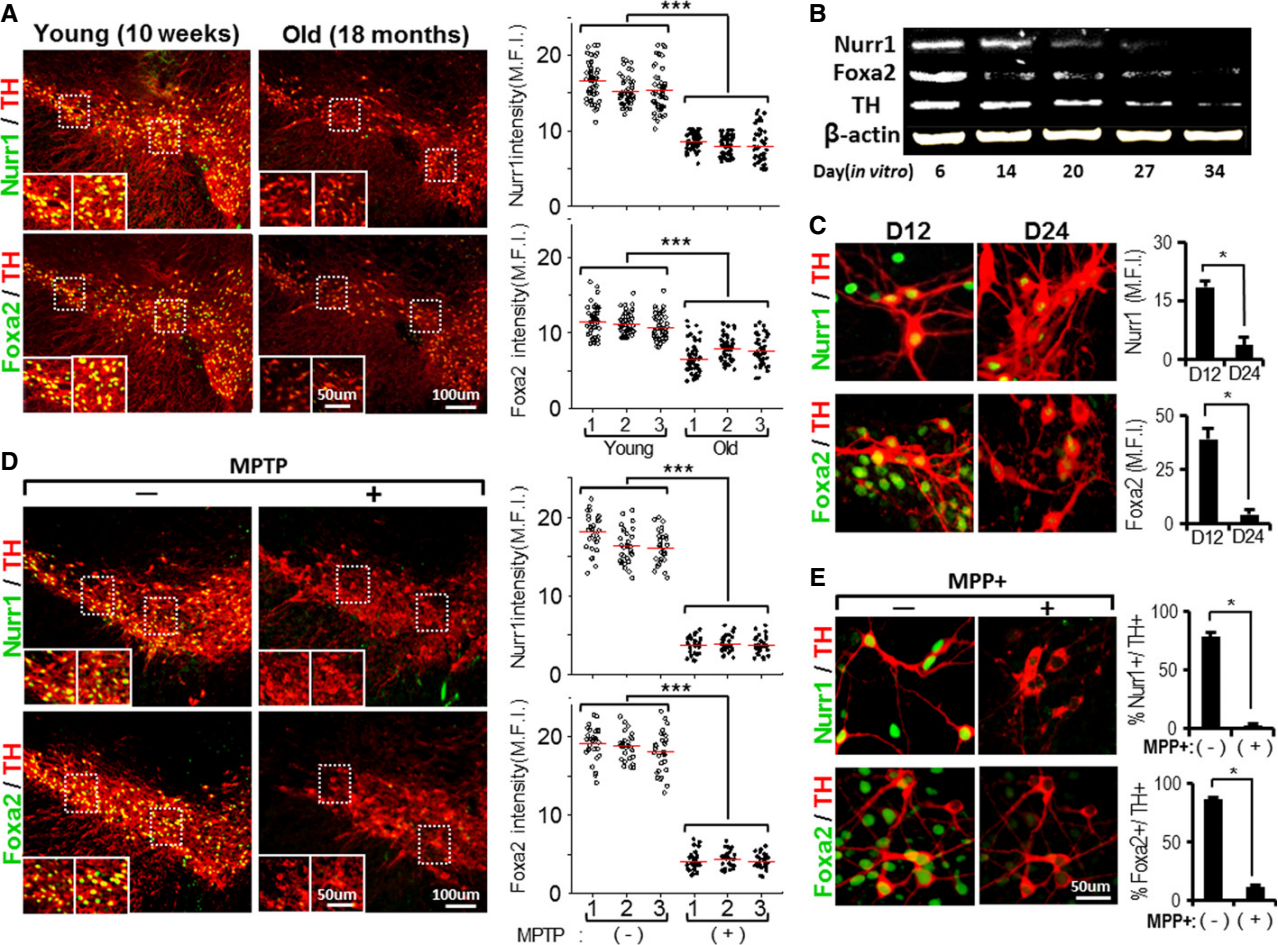
Reduction of Nurr1 and Foxa2 in mDA neurons during aging and degeneration

Aging is a crucial predisposing factor for PD (Moghal *et al*, 1994). Interestingly, both Nurr1 and Foxa2 levels were significantly lower in the individual mDA neurons of old mice (18 months) than in their young counterparts (10 weeks) (Fig2A). In agreement with this, Nurr1 and Foxa2 levels also gradually declined in cultured mDA neurons during the late period of culture (6–34 days after onset of differentiation *in vitro*; Fig2B), ultimately giving rise to abundant TH^+^ cells negative for Nurr1/Foxa2 (% Nurr1^+^ of TH^+^ cells: 90.5% at D12 versus 14.3% at D24 and %Foxa2^+^ of TH^+^ cells: 91.3% at D12 versus 12.9% at D24, Fig2C). These findings are consistent with the decreased Nurr1 levels in the SN of elderly persons (Chu *et al*, 2002) and also imply that loss of Foxa2 is an additional aspect of the aging process in mDA neurons.

A–C Nurr1 and Foxa2 protein levels in mDA neurons decrease in the midbrains of old mice *in vivo* (A) and *in vitro* after long-term culture (B and C). (A) Nurr1 and Foxa2 protein levels were compared in individual mDA neurons of the midbrains of young (10 weeks) and old mice (18 months) of the same mouse strain (C57BL/6, male). All the midbrain sections were immunofluorescently co-stained with Nurr1/TH (upper) and Foxa2/TH (lower) under identical conditions, and levels of Nurr1 and Foxa2 proteins were determined in individual TH^+^ mDA neurons by measuring mean fluorescence intensities (MFI) using LAS image analysis (Leica). Dots in the graphs represent the Nurr1 and Foxa2 MFI values of individual TH^+^ DA neurons in the SN of each animal. The average MFI values (indicated by horizontal lines) of three animals from each group were compared (***P = 5.25E-88 for Nurr1 intensity, 5.57E-40 for Foxa2 intensity, one-way ANOVA followed by Bonferroni *post hoc* test). Nurr1 and Foxa2 protein levels were also quantified in cultured mDA neurons over 6–34 days *in vitro* by Western blotting (B) and by immunocytochemical analysis (C). Significantly lower MFI values on day 24 of culture (D24) compared to D12 at *P = 0.027 (Nurr1), *P = 0.012 (Foxa2), *n* = 60–70 TH^+^ cells from two cultures in each group, unpaired Student's *t*-test.

D, E Loss of Nurr1 and Foxa2 expression in mDA neurons after treatment with the neurotoxin MPTP (or MPP+). Mice (10 weeks old) were treated with MPTP for 5 days as described in Materials and Methods. Three days after the last MPTP injection, Nurr1 and Foxa2 protein levels in the TH^+^ mDA neurons of the MPTP-treated SN were compared with in the mDA neurons of untreated mice (D) (***P = 5.47E-103 for Nurr1 intensity, 1.53E-111 for Foxa2 intensity, one-way ANOVA followed by Bonferroni *post hoc* test.). The effects of neurotoxin treatment were also determined in mDA neuron cultures treated with MPP+ (250 μM, 8 h, E). *P = 0.015 (% Nurr1^+^/TH^+^ cells), *P = 0.018 (% Foxa2^+^/TH^+^ cells), unpaired Student's *t*-test.

1-Methyl-4-phenyl-1,2,3,6-tetrahydropyridine (MPTP) and its active metabolite 1-methyl-4-phenylpyridinium (MPP+) specifically induce mDA neuron degeneration. A decline in Nurr1 and Foxa2 protein levels was evident in TH^+^ mDA neurons of mice (10 weeks old) after MPTP treatment (Fig2D). The toxin-induced loss of Nurr1 and Foxa2 was also seen in TH^+^ mDA neurons *in vitro* before any obvious loss of mDA neurons was detected (Fig2E). These findings taken together indicate that decrease in Nurr1 and Foxa2 expression is a manifestation of cellular aging and degeneration of mDA neurons, and thus, forced expression of these factors might be a therapeutic option for protecting mDA neurons against degenerative processes in PD.

### Forced Nurr1 and Foxa2 expression protects mDA neurons against toxic insults in mDA neuron–glia cultures

**Figure 3:**
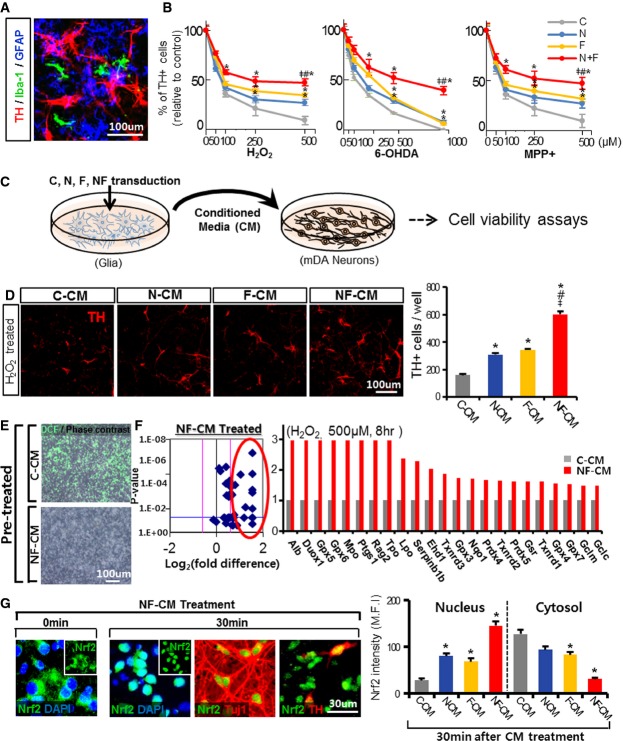
Forced expression of Nurr1 and Foxa2 protects mDA neurons from toxic insults *in vitro*

To test the therapeutic potential of forced Nurr1/Foxa2 expression, we used primary mDA neuron–glia cultures derived from the mouse VM (Fig3A), which reflect the *in vivo* cellular composition and environment of the midbrain. The cultures were transduced with lentiviruses expressing Nurr1, Foxa2, Nurr1 + Foxa2, or control empty vector (control). Numbers of DA neurons 5 days after transduction (days *in vitro*, DIV 15) were significantly increased in the Nurr1- or Foxa2-transduced cultures. The number of viable DA neurons was the greatest after transduction of Nurr1 + Foxa2 (control, 1,136 ± 819; Nurr1, 1,533 ± 19; Foxa2, 1,766 ± 21; Nurr1 + Foxa2, 1,939 ± 22 cells/well, *n* = 4 culture wells in each group). Numbers of TH^+^ DA neurons decreased with increasing H_2_O_2_ dose. Introduction of Nurr1 and Foxa2 additively or synergistically prevented the H_2_O_2_-induced loss of TH^+^ cells (Fig3B, left). Nurr1 and Foxa2 exerted a similar protective effect against the parkinsonism toxins MPP^+^ and 6-hydroxydopamine (6-OHDA) (Fig3B, middle and right).

A Representative image for TH^+^ (mDA neuron), GFAP^+^ (astrocyte), and Iba-1^+^ (microglia) cells in mDA neuron–glia cultures used in the gain-of-function experiments.

B mDA neurons in the cultures transduced with Nurr1 and/or Foxa2 are resistant to toxic stimuli induced by H_2_O_2_ (50–500 μM), 6-OHDA (50–1,000 μM), or MPP+ (50–500 μM). The mDA neuron–glia cultures were transduced with lentiviruses expressing Nurr1 (N), Foxa2 (F), Nurr1 + Foxa2 (NF), or control (C) and treated with the toxin for 8 h. Viable TH^+^ cells were counted on the following day. Shown in the graphs are percent TH^+^ cells relative to the respective toxin-untreated cultures. TH^+^ cells of C, N, F, and NF were compared at the same concentrations of the toxins. Significantly different from the control (C)*, from N^#^, and from F^‡^ at *P* < 0.05, *n* = 10 cultures each; one-way ANOVA followed by Bonferroni *post hoc* test.

C, D Forced expression of Nurr1 and Foxa2 in glia exerts neuroprotective roles on mDA neurons in a paracrine mode. Experimental scheme (C) to test the effects of paracrine factors released from Nurr1-/Foxa2-expressing glia. Mixed astrocytes + microglia cultures (derived from VM tissue of mouse pups on postnatal day 1) were transduced with N, F, NF, or C, and conditioned media (CM) were prepared from the transduced glia and added to mDA neuron cultures. Two days after the CM treatment, H_2_O_2_ (500 μM, 8 h)-mediated cell death was measured by counting viable TH^+^ cells (D). Significantly different from the control (C-CM)*, N-CM^#^, F-CM^‡^ at *P *<* *0.05. *P*-values: 0.031 (N-CM*), 0.021 (F-CM*), 0.017 (NF-CM*), 0.022 (NF-CM^#^), and 0.027 (NF-CM^‡^); one-way ANOVA followed by Bonferroni *post hoc* test.

E–G NF-expressing glia secrete factors that reduce oxidative stress by inducing Nrf2-mediated antioxidant gene expressions. Oxidative stress measured by DCF (oxidative stress indicator) staining (E). Primary cultured mDA neurons were pre-treated with C-CM (upper) or NF-CM (lower) for 2 days and then exposed to H_2_O_2_ (250 μM) in the presence of the CM. Four hours later, DCF staining was carried out. (F) Expression array for antioxidant genes. mDA neuron cultures were pre-treated with C-CM, N-CM, F-CM, or NF-CM for 2 days before exposure to 500 μM H_2_O_2_ for 8 h, and mRNA expression levels of 39 antioxidant genes were estimated using an RT^2^ PCR Profiler Array^R^. Volcano plot (left) demonstrating a tendency for increased antioxidant gene expression in mDA neuron cultures with NF-CM treatment. The pink lines indicate the threshold of 1.5-fold changes in gene expression. The 23 antioxidant genes up-regulated 1.5-fold (circled) by NF-CM treatment relative to C-CM treatment are listed on the bar graph (right). (G) NF-CM treatment of mDA neurons activates cytosolic Nrf2 proteins (0 min) by inducing nuclear translocation (30 min). Shown in the graph are cytosolic and nuclear Nrf2 protein levels (MFI) 30 min after C-CM, N-CM, F-CM, and NF-CM treatments. *P < 0.05, *n* = 50–60 cells in each group. *P*-values: 0.032 (N-CM*), 0.029 (F-CM*), 0.019 (NF-CM*) for the Nrf2 intensity in nucleus, 0.029 (F-CM*), 0.039 (NF-CM*) for the Nrf2 intensity in cytosol; one-way ANOVA followed by Bonferroni *post hoc* test.

### Glia expressing Nurr1 + Foxa2 secrete molecules that protect mDA neurons via Nrf2-mediated anti-oxidation

Most previous works (Le *et al*, 1999; Kadkhodaei *et al*, 2009, 2013; Volakakis *et al*, 2010; Malewicz *et al*, 2011; Decressac *et al*, 2012) have suggested that Nurr1 and Foxa2 in mDA neurons exert cell-autonomous cell survival/protective effects, and this view is supported by the knockdown experiments shown in Fig1G. However, in agreement with previous studies (Saijo *et al*, 2009), lentivirus-mediated transgene expression was detected in only a few TH^+^ DA neurons, but in many more GFAP^+^ or Iba-1^+^ glial cells, indicating that the cytoprotective effects observed in our gain-of-function experiments, at least, were not mediated by cell-autonomous actions of Nurr1 and Foxa2, but probably in an extrinsic manner by Nurr1- and Foxa2-expressing glia. This hypothesis is consistent with the study of Saijo *et al* (2009), demonstrating a paracrine neuroprotective effect of Nurr1. To test for a potential paracrine action, we transduced glia (astrocytes + microglia derived from midbrains) with Nurr1, Foxa2, Nurr1 + Foxa2, or control, and medium conditioned in the glia was added to primary mDA neuron cultures (Fig3C). Conditioned medium (CM) from Nurr1-transduced glia (Nurr1-CM) and Foxa2-transduced glia (Foxa2-CM) exerted a neuroprotective effect (Fig3D). The neuroprotective effect by the CM prepared from Nurr1 + Foxa2-transduced glia (Nurr1 + Foxa2-CM) was more dramatic than those of Nurr1-CM and Foxa2-CM.

Levels of ROS in H_2_O_2_-treated mDA neuron cultures were greatly reduced by pre-treatment with Nurr1 + Foxa2-CM (Fig3E). In a super-array analysis, the patterns of antioxidant gene expression in the cultures treated with Nurr1-CM or Foxa2-CM were not greatly different from the cultures treated with control-CM (Supplementary Fig S2). However, expression of most of the antioxidant genes tested was increased by Nurr1 + Foxa2-CM (Fig3F), indicating that the combined expression of Nurr1 +  Foxa2 in glia is required to reduce oxidative stress in neighboring mDA neurons by inducing antioxidant gene expression. Nuclear factor-erythroid 2-related factor 2 (Nrf2) is a master regulator of antioxidant defense via the scavenging of ROS, which acts by inducing the expression of antioxidant genes. Upon activation, cytoplasmic Nrf2 proteins are translocated to the nucleus where they bind to the antioxidant responsive element (ARE) and activate transcription of a large array of antioxidant genes (for review, see Kensler *et al*, 2007). Nurr1 + Foxa2-CM treatment rapidly and robustly triggered the nuclear localization of Nrf2 in mDA neuron cultures (Fig3G).

### Molecules responsible for the paracrine neuroprotective actions of Nurr1 and Foxa2

**Figure 4:**
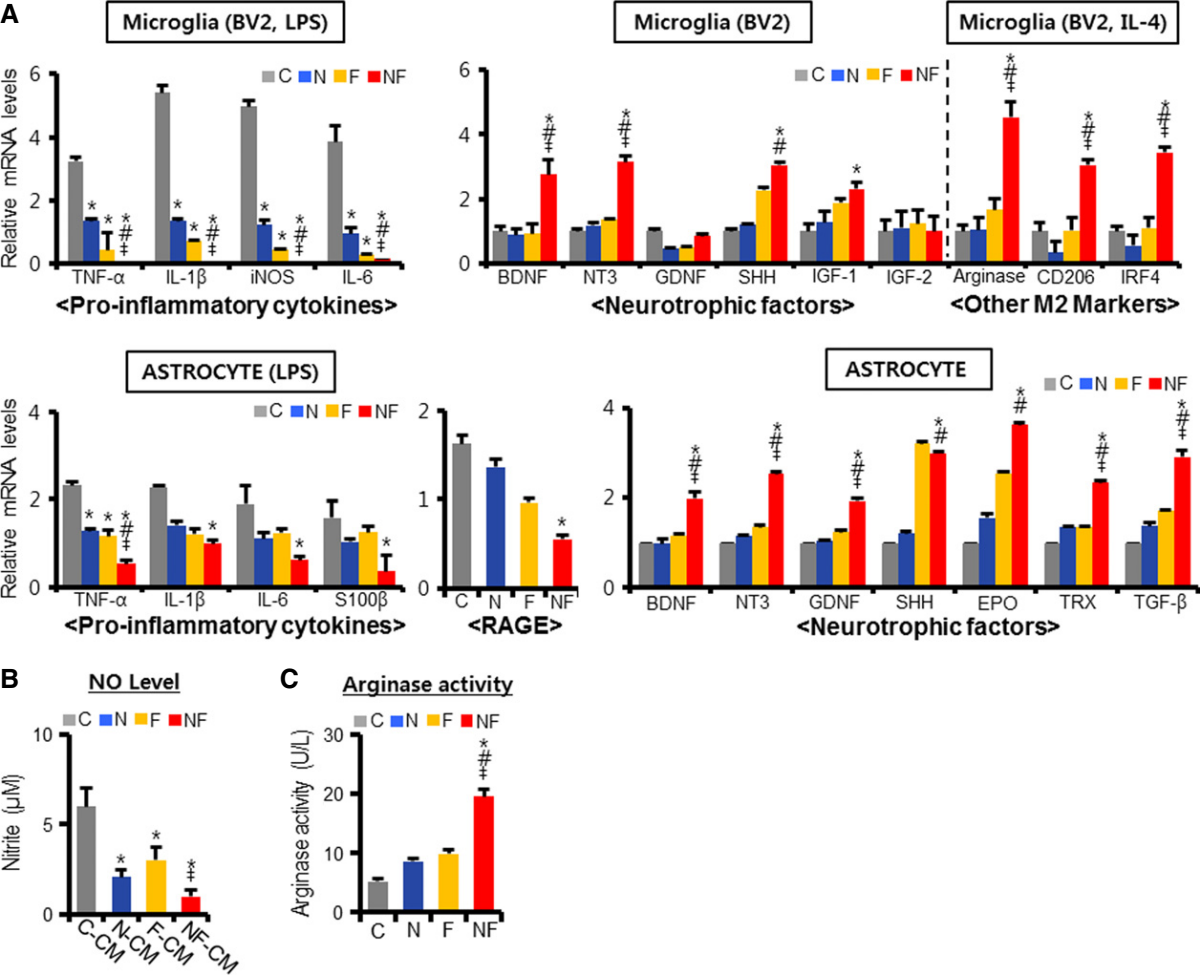
Mechanisms for the paracrine neuroprotective roles by Nurr1- and Foxa2-expressing glial cells

We next sought to identify the paracrine factors responsible for the Nurr1- and Foxa2-mediated neuroprotection. Saijo *et al* (2009) have reported that knockdown of Nurr1 aggravated mDA neuronal death by increasing the production/release of pro-inflammatory cytokines from activated microglia. Consistent with this, we found that forced expression of Nurr1 in BV2 microglia led to a significant reduction in the expression of the pro-inflammatory cytokines, tumor necrosis factor-α (TNF-α), inducible nitric oxide (NO) synthase (iNOS), and interleukin-1β (IL-1β) upon exposure to the Toll-like receptor 4 (TLR4) ligand, lipopolysaccharide (LPS) (Fig4A, upper). Interestingly, a decrease in pro-inflammatory cytokine expression was also evident in Foxa2-expressing microglia, indicating that Foxa2 alone, without interacting with Nurr1, can exert an anti-inflammatory action. Regulation of inflammatory responses by Foxa2 has also been shown in other tissues (Lappalainen *et al*, 2005; Tang *et al*, 2013). Importantly, Nurr1 and Foxa2 synergism was very dramatic, and transcripts of the pro-inflammatory cytokines were undetectable or barely detected after LPS treatment of Nurr1 + Foxa2-expressing BV2 cells (Fig4A, upper). In line with the gene expression results, NO levels in the CM from the Nurr1 + Foxa2-transduced microglia were lower than in the CM from control microglia (Fig4B). As well as microglia, astrocytes can create an inflammatory environment by releasing pro-inflammatory molecules (van Noort & Bsibsi, 2009; Gorina *et al*, 2011), and similar patterns of pro-inflammatory cytokine expression decrease were seen in primary astrocytes transduced with Nurr1 and Foxa2 (Fig4A, lower). S100β, a soluble protein released from astrocytes, acts as a damage-associated factor via receptor for advanced glycation end products (RAGE) pathways (Hofmann *et al*, 1999; Villarreal *et al*, 2011). Previous studies have shown that both S100β and RAGE increase in PD patients and MPTP-treated mice, and contribute to the damage to mDA neurons (Muramatsu *et al*, 2003; Sathe *et al*, 2012). We found that Nurr1 + Foxa2 expression in astrocytes significantly decreased S100β and RAGE levels (Fig4A, lower).

A Real-time PCR analysis for pro-inflammatory cytokines and neurotrophic factors. BV2 microglia and primarily cultured astrocytes were transduced with C, N, F, and NF. Real-time PCR analyses were carried out 12 h after LPS (100 ng/ml, for pro-inflammatory cytokine expressions), IL-4 (10 ng/ml, other M2 markers), or without the treatment (neurotrophic factors). Significantly different from the control (C)*, N^#^, F^‡^ at *P* < 0.05, *n* = 3–6 PCRs; one-way ANOVA followed by Bonferroni *post hoc* test.

B, C NO levels released (B) and arginase activity (C) were measured in the media and cells for BV-2 microglia cultures, respectively. Significantly different from the control (C)*, N^#^, F^‡^ at *P *<* *0.05, *n* = 3 reactions, *P*-values: 0.027 (N-CM*), 0.033 (F-CM*), 0.018 (NF-CM*), and 0.033 (NF-CM^‡^) for the NO level, 0.011*, 0.025^#^, and 0.036^‡^ for the arginase activity; one-way ANOVA followed by Bonferroni *post hoc* test.

Glial cells can be polarized into alternative phenotypes (the “alternative” M2 phenotype), versus the detrimental M1 phenotype resulting from classical activation. The M2 phenotype can create a beneficial environment for neurons by secretion of neurotrophic factors such as neurotrophin 3 (NT3), brain-derived neurotrophic factor (BDNF), glial cell-derived neurotrophic factor (GDNF), erythropoietin, thioredoxin, transforming growth factor-β (TGF-β), sonic hedgehog (SHH) from astrocytes (Trendelenburg & Dirnagl, 2005), and insulin-like growth factor 1/2 (IGF1/2) and BDNF from microglia (Suh *et al*, 2013). The observation of Nrf2 activation and expression of antioxidant genes in mDA neurons after Nurr1 + Foxa2-CM treatment (Fig3F and G) suggest that the Nurr1- and Foxa2-mediated paracrine actions involve the release of neurotrophic factors, because these factors are major cytokines activating Nrf2 via the MAPK and PI3K pathways (for review, see Lee & Johnson, 2004; Weissmiller & Wu, 2012). Indeed, primary astrocytes transduced with Nurr1 and/or Foxa2 expressed significantly elevated levels of mRNAs for the trophic factors BDNF, NT3, GDNF, SHH, erythropoietin, thioredoxin, and TGF-β (Fig4A, lower). The effect of the combination of Nurr1 + Foxa2 on expression of BDNF, NT3, SHH, and IGF-1 was also evident in BV2 microglia (Fig4A, upper). The anti-inflammatory cytokines IL-4/IL-13 trigger M2 polarization of microglia by inducing arginase 1, CD206, and interferon regulatory factor 4 (IRF4) (Hu *et al*, 2012). Upon IL-4 treatment, the expression of M2 markers (Fig4A), as well as arginase enzyme activity (Fig4C), was greatly increased in Nurr1 + Foxa2-transduced BV2 cells. These findings suggest that Nurr1 and Foxa2 in glia additively/synergistically exert neuroprotective effects on mDA neurons via two paracrine pathways: (i) decreasing the production and release of pro-inflammatory cytokines and (ii) enhancing the synthesis and secretion of neurotrophic factors.

### Therapeutic potential of AAV-mediated gene delivery of Nurr1 and Foxa2 in the mouse model of PD

**Figure 5:**
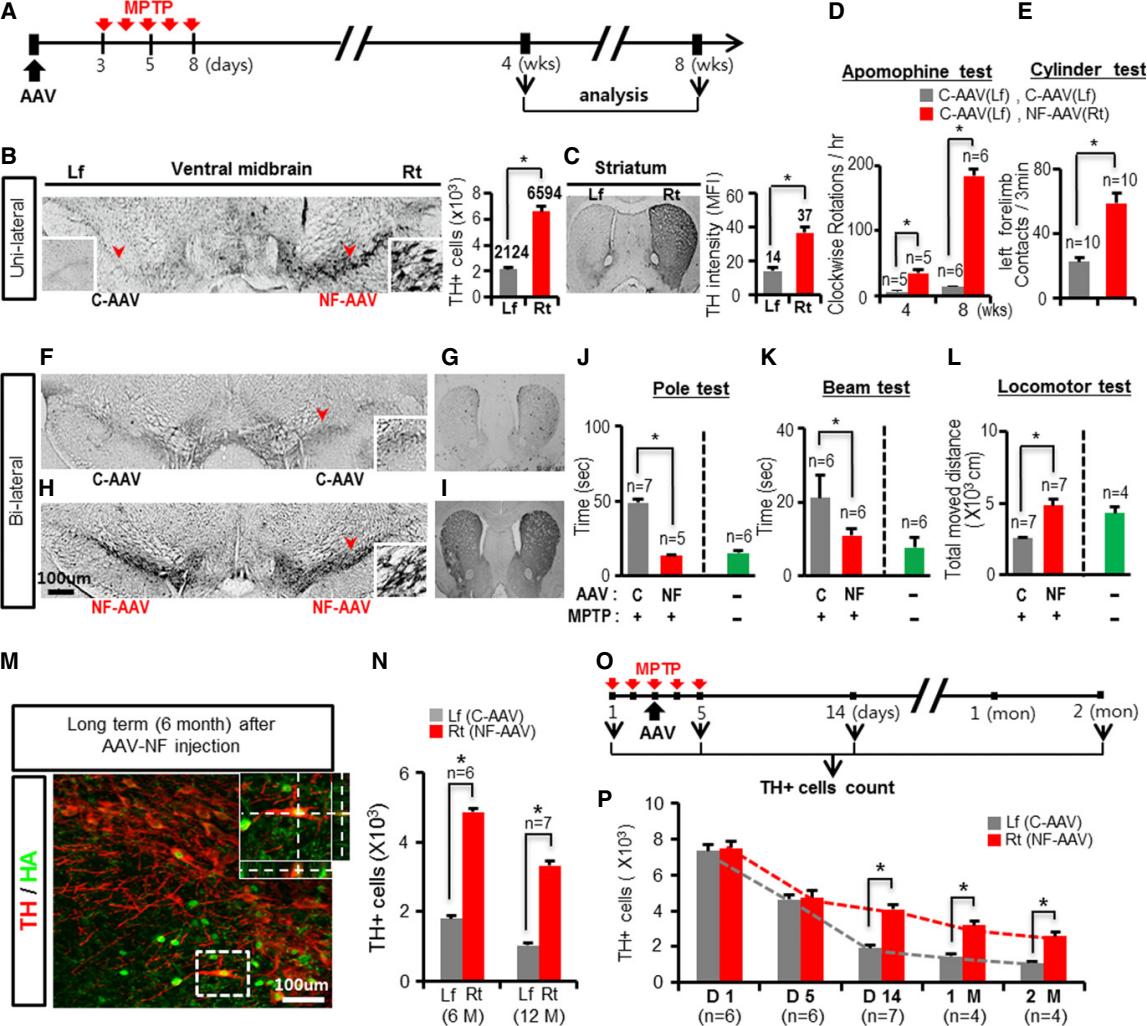
Therapeutic effects of AAV-mediated gene delivery of Nurr1 + Foxa2 in the mouse PD model

Based on our *in vitro* data, we examined whether forced expression of Nurr1 and Foxa2 could forestall degeneration of mDA neurons in PD. To this end, we chose the best-characterized MPTP mouse PD model (Beal, 2001) using a subchronic systemic approach (MPTP injection of 30 mg/kg, i.p., for five consecutive days) and the adeno-associated viral (AAV) system for gene delivery (schematized in Fig5A and O). Because of AAV's low immunogenicity, lack of cytotoxic response, and ability to infect non-dividing cells, clinical trials using it are currently underway in many disorders including PD (for reviews, see McCown, 2011; Weinberg *et al*, 2013). By injecting green fluorescent protein (GFP)-expressing AAVs into mouse midbrains, we confirmed expression of the transgene in our target cells expressing TH (DA neurons), GFAP (astrocyte), and Iba-1 (micro-glia) (Supplementary Fig S3).

A Experimental scheme. As shown, PD model mice were generated by IP injection of MPTP, and AAVs expressing Nurr1 and Foxa2 (NF-AAV) or AAVs expressing empty vector (control, C-AAV) were stereotaxically injected 3 days prior to the initial MPTP treatment.

B–E Cytoprotective and behavioral effects of NF expression were assessed in PD mice unilaterally injected with NF-AAVs on the right side of midbrains. C-AAVs were injected into the left midbrains of the same mice. TH^+^ mDA neuron numbers in the midbrain (B) and TH^+^ fiber intensities in the striatum (C) of the NF-AAV-injected right sides were compared with those of the corresponding left sides after 8 weeks. Insets of (B), high-powered images of the areas indicated by red arrowheads in the C-AAV (left) and NF-AAV-injected (right) sides. TH^+^ cell numbers, *P =* *0.000078, *n* = 7, paired Student's *t*-test. The sectioned striatal tissues were immunofluorescently stained for TH, and TH^+^ fiber intensities were estimated from mean fluorescence intensities (MFI). *n* = 56 microscopic fields at a magnification of 200×  each for the left and right striatum. *P =* *0.000003, paired Student's *t*-test. Behavioral asymmetry in the mice unilaterally injected with NF-AAVs (right side) was assessed using the apomorphine-induced rotation test (D) after 4 and 8 weeks. The cylinder test (E) was carried out after 8 weeks to determine left forelimb movement. As a control, the behaviors were tested in PD mice bilaterally injected with C-AAV. *P =* *0.028 (rotation test), 0.043 (cylinder test); one-way ANOVA followed by Bonferroni *post hoc* test.

F–L Behaviors of the PD mice injected with NF-AAVs bilaterally at both sides of the midbrain were assessed using the pole (J), beam (K), and locomotor (L) tests. *P =* *0.004 (pole), 0.044 (beam), and 0.045 (locomotor); one-way ANOVA followed by Bonferroni *post hoc* test. Shown in (F–I) is a representative TH-stained midbrain (F, H) and striatum (G, I) of mice bilaterally injected with C-AAVs (F, G) and NF-AAVs (H, I).

M, N Sustained mDA neuroprotective effects. Shown in (M) is a confocal image of TH/HA-stained cells 6 months after NF-AAV injection (Foxa2 gene tagged with HA). Inset, *z*-stacked image of the boxed area along the *y*-axis (right) and *x*-axis (lower). TH^+^ cells in the right (NF-AAV) and left (C-AAV injected) midbrains were counted 6 and 12 months after AAV injection (N). *P =* *0.000019 (6M), 0.000066 (12M), *n* = 6–7, paired Student's *t*-test.

O, P NF-AAV-mediated mDA neuroprotective effects were further assessed with the experimental schedule (O), in which AAV injections were subjected after three consecutive MPTP injections. Shown in (P) is TH^+^ cell counts of the left and right sides of the midbrains. *P =* *0.000012 (D14), 0.000036 (1M), 0.000027 (2M), *n* = 6–7, paired Student's *t*-test.

**Figure 6:**
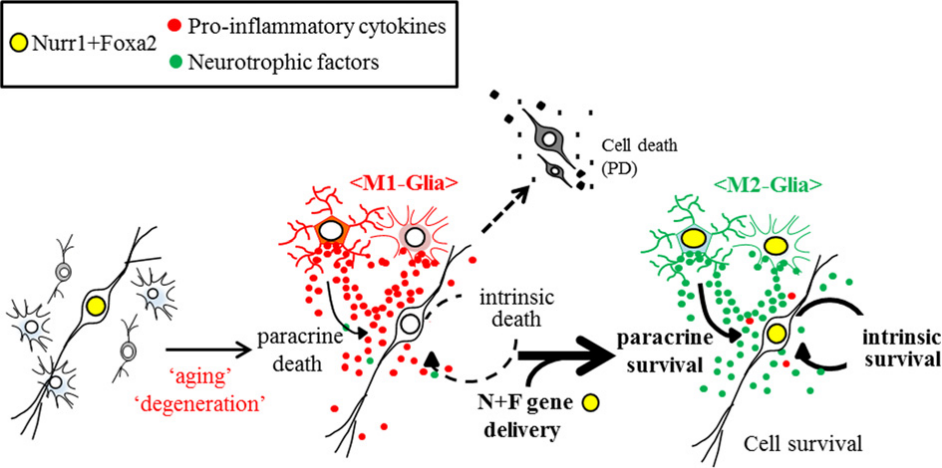
Schematic representation of mDA neuron survival promoted by forced Nurr1 + Foxa2 gene delivery Nurr1 and Foxa2 are endogenously expressed in mDA neurons and promote cell survival in a cell-autonomous manner (left). Expression of Nurr1 and Foxa2 is reduced in mDA neurons during aging and degenerative processes. In addition, glia come to be polarized toward the harmful M1-type. DA neurons with reduced Nurr1/Foxa2 expression are more vulnerable to the toxic environment in the aged or degenerating midbrain (middle). Forced expression of Nurr1 + Foxa2 in mDA neurons promotes their survival. In addition, Nurr1 + Foxa2 transgene expression in glial cells (M2-type) creates a neuroprotective environment by suppressing the secretion of pro-inflammatory cytokines as well as promoting neurotrophic factor release (right).

Intraperitoneal injection of MPTP leads to a gradual but massive loss of DA neurons in the midbrain: approximately 80–90% of TH^+^ mDA neurons have been lost 2 months after MPTP injection. DA neuronal losses on the two sides were not significantly different. In this bilateral PD model, AAVs expressing Nurr1 and/or Foxa2 were injected into one side (right) of each midbrain (3 days prior to MPTP treatment), whereas the other side (left) was injected with control-AAVs carrying empty vector. The cytoprotective effect by injection of AAVs expressing the combination of Nurr1 + Foxa2 was very dramatic, consistent, and reproducible. Without exception, TH^+^ DA neuron numbers on the Nurr1 + Foxa2-AAV-injected side were greater than those on the control side (at least > 2.6-fold, *n* = 7, *P* = 0.000078, paired *t*-test) (Fig5B and Supplementary Fig S4A), and there was a similar difference in Nissl-stained and NeuN^+^ neuronal numbers between the Nurr1 + Foxa2-AAV- and control-AAV-injected sides (Supplementary Fig S5). The average number of TH^+^ DA neurons after 2 months was 6,594 cells on the Nurr1 + Foxa2-AAV-injected side versus 2,124 cells on the control side. Based on the average TH^+^ cell number counted in MPTP-untreated midbrains (8,143 cells), our TH^+^ cell counts indicate that 69% of mDA neurons were rescued from MPTP-induced degeneration by Nurr1 + Foxa2-AAV. Injection of Nurr1-AAV or Foxa2-AAV alone had much less effect than the combined treatment (Supplementary Fig S4A). In accord with the *in vitro* findings shown in Fig4A, the transcript and protein levels of the cytotoxic mediators S100β and GFAP were greatly reduced on the Nurr1 + Foxa2-AAV-injected sides of the midbrain (Supplementary Fig S6A and B). In addition, mRNA levels of the pro-inflammatory cytokines IL-1β and iNOS were significantly lower in the Nurr1 + Foxa2-AAV-injected sides than those of the control sides, while the expressions of BDNF, NT3, SHH, and TGF-β were greater in the Nurr1 + Foxa2-AAV-injected sides (Supplementary Fig S6C). Nigrostriatal DAergic innervation was also substantially protected on the Nurr1 + Foxa2-AAV-injected side, as shown by the TH^+^ fiber intensities in the striatum (mean fluorescent intensity (MFI): 37 on the Nurr1 + Foxa2-AAV injected versus 14 on the control side) (Fig5C and Supplementary Fig S4B). Consequently, the mice displayed massive rotatory behaviors toward the Nurr1 + Foxa2-AAV-injected side (right, clockwise) when the DA receptor agonist apomorphine was i.p. injected (Fig5D). Furthermore, when locomotor activity was estimated by the cylinder test 8 weeks after MPTP treatment, the mice injected with Nurr1 + Foxa2-AAV on the right side (control-AAV on the left side) used their left forelimbs more often when rearing against a wall than did the mice injected with control-AAV on both sides (Fig5E), while the numbers of right forelimb contacts were not significantly different between the groups (46.5 ± 6.9 versus 50.1 ± 5.7/3 min).

We further addressed several practical issues in PD therapy. First, we examined the effects of bilateral Nurr1 + Foxa2-AAV injection on the behaviors of MPTP-treated mice, because both sides of the SN are commonly affected in PD patients. Along with its strong protective effects on mDA neurons and striatal DA fiber innervation (Fig5F–I), bilateral Nurr1 + Foxa2-AAV injection substantially protected the behaviors assessed by the pole (Fig5J), beam (Fig5K), and locomotor (Fig5L) tests. Another issue is maintenance of the therapeutic effect. AAV-delivered transgene expression was sustained for up to 6 months (Fig5M), and Nurr1 + Foxa2-mediated cytoprotective effects were evident 1 year after gene delivery (Fig5N). In most cases, treatment starts in PD patients undergoing progressive degenerative process. We therefore examined the effect of Nurr1 + Foxa2-AAV on midbrains already undergoing degeneration. To do this, Nurr1 + Foxa2-AAVs were injected 2 days after the initial injection of MPTP (Fig5O). Around 40% of mDA neurons in the midbrains were lost during days 1–5 after MPTP treatment, and TH^+^ cell numbers on the right side (Nurr1 + Foxa2-AAV injected) were not different from those on the left side (C-AAV injected) on day 5 after MPTP treatment (Fig5P), probably because expression of the exogenous Nurr1 and Foxa2 from the AAVs takes several days. DA neuron numbers on the left side declined greatly by days 5–14 (from 4,629 cells on day 5 to 1,904 cells on day 14; 2,725 mDA neuron lost). By contrast, 4,056 TH^+^ mDA neurons on the Nurr1 + Foxa2-AAV-injected right side of the midbrain survived on day 14, resulting in the loss of only 682 mDA neurons on days 5–14. The Nurr1 + Foxa2-mediated neuroprotective effect was further detected up to 2 months (Fig5P). Based on these findings, we propose that AAV-mediated Nurr1 + Foxa2 gene delivery is a promising therapeutic tool in PD.

## Discussion

Current symptomatic treatments of PD cannot halt disease progression. To overcome this limitation, disease-modifying strategies are being sought. Discouraging results have been obtained in clinical trials of neurotrophic factors in the treatment of neurologic disorders (Thoenen & Sendtner, 2002). While there are many possible reasons for these failures, there is a consensus that the delivery of cytokines and chemicals to their desired neuronal targets within the central nervous system is a challenge. Even local administration of trophic factors directly to the vicinity of the desired site appeared to give discouraging outcomes due to inefficient diffusion to the target site (Thoenen & Sendtner, 2002; Sherer *et al*, 2006). Furthermore, adverse side effects can be expected from systemic administration and/or broad-spectrum action of candidate agents. These difficulties prompt an alternative approach involving manipulating factors associated with neurotrophic or cytoprotective processes in mDA neurons at the gene levels, for example, by exploiting transduction by viral vectors.

In the current study, we examined the combined expression of Nurr1 and Foxa2 as a therapeutic tool for PD. The rationale for this was as follows. First, both factors are expressed in the mDA neurons of the adult SN and interact to promote the survival of these neurons and protect them against toxic insults. However, the levels of these factors decrease during aging and degeneration. Forced expression of the genes, which are associated with target functions in normal physiology, but lost in pathologic states, would be an appropriate option for the therapeutic development. Genomewide profiling studies have demonstrated that PD pathology is associated with broad transcriptional dysregulation (Cooper-Knock *et al*, 2012). Thus, the decreased Nurr1 and Foxa2 levels observed could be interpreted as part of the context of PD pathology. α-Synuclein, clumps of which are hallmarks of PD, has been suggested as an important cause of the perturbation of gene transcription in PD (McLean *et al*, 2000; Specht *et al*, 2005; Decressac *et al*, 2012). Specifically, α-synuclein accumulation is associated with the down-regulation of the transcription factors critical for neuronal survival, including Nurr1, myocyte enhancer factor-2D, DNA methyltransferase 1, and the transcription factor EB (Chu *et al*, 2011; Desplats *et al*, 2011; Decressac *et al*, 2012, 2013a). In addition, other factors related to oxidative stress and inflammatory reactions may directly or indirectly influence protein levels via protein degradation (Jo *et al*, 2009; Lin *et al*, 2012).

Second, the strategy proposed in this study would be less hazardous as it is without tumorigenic side effects. Gene therapy with molecules involved in survival pathways, for example, Akt-mTOR, has been suggested for PD (Ries *et al*, 2006; Kim *et al*, 2011, 2012). However, most of these signaling molecules are involved in a broad spectrum of cellular events, including the cell cycle. By contrast, Nurr1 and Foxa2 are rather specific for the development, maintenance, and survival of mDA neurons in the midbrain, and their tumorigenic potential is expected to be quite low, given the strong cell cycle arrest induced by their overexpression (Castro *et al*, 2001; Lee *et al*, 2010; Liu *et al*, 2012).

Third, in addition to their protective roles in mDA neurons, forced expression of Nurr1 and Foxa2 in glial cells shifts the diseased environment surrounding mDA neurons in the midbrain toward a therapeutic one by reducing pro-inflammatory cytokine levels as well as by increasing neurotrophic cytokine release. Because of this, the combined expression of Nurr1 + Foxa2 could become an ideal and prospective therapeutic approach, and one superior to strategies involving exclusively intrinsic or extrinsic modes of action. Epigenetic regulation by Nurr1 and Foxa2 may possibly involve the regulation of the pro-inflammatory/trophic factor expression. In addition to the trans-repressive role of Nurr1 (Saijo *et al*, 2009), we have shown that Nurr1 and Foxa2 activate gene transcription epigenetically by opening chromatin structures by histone acetylation and methylation (Yi *et al*, 2014). Consistent with this, histone deacetylase (HDAC) inhibitors increase neurotrophic factor release from glia cells (Peng *et al*, 2005; Chen *et al*, 2006, 2012; Wu *et al*, 2008). Furthermore, the H3K27me3 histone demethylase, Jumonji domain containing 3 (jmjd3), is essential for microglia polarization toward the M2 type expressing neurotrophic factors (Tang *et al*, 2014), and jmjd3 mRNA in BV2 microglia was up-regulated by the combined Nurr1 and Foxa2 expression (Supplementary Fig S7).

AAVs are very poorly immunogenic and thus persist for years as extrachromosomal episomes. AAVs (serotype 2) are predominantly being used in current gene therapeutic trials, because of their proven efficacy and safety (for reviews, see McCown, 2011; Terzi & Zachariou, 2008). Thus with a clinical trial in mind, we used the AAV vector system for Nurr1 and Foxa2 gene delivery into the PD mouse SN. We showed that AAV-mediated transgene expression was efficiently induced in the midbrain SN over a prolonged period (at least 6 months) and that combined Nurr1 + Foxa2 transgene expression strongly protected mDA neurons from MPTP neurotoxin and restored behaviors associated with nigrostriatal DA neurotransmission. The neuroprotective effect of Nurr1 + Foxa2-AAV gene delivery was not only dramatic (ca 70% of mDA neurons were protected from MPTP-induced neurodegeneration), but also completely reproducible: Numbers of surviving TH^+^ DA neurons in the SN injected with Nurr1 + Foxa2-AAV were always far greater than in the control SN. AAV-induced transgene expression was detected in all types of cells in the midbrain—neurons, astrocytes, and microglia—in our specific experimental conditions. However, consistent with the acknowledged neurotropism of AAVs (reviewed in McCown *et al*, 1996), AAV-mediated transgene expression was preferentially detected in mDA neurons, while the proportion of glial cells expressing the transgene was not high: 1 week after GFP-expressing AAV injection, 7.2 ± 0.9% and 1.4 ± 0.3% out of total GFP^+^ cells in the SN regions were positive for GFAP and Iba-1, respectively, while 66.4 ± 4.6% of GFP^+^ cells expressed TH (*n* = 5 sections). The development of vector systems with better glial tropism may be required to improve the ability of Nurr1 + Foxa2 to create a neuroprotective environment surrounding degenerating mDA neurons. Despite the observed low infectivity of glia, a significant anti-inflammatory change (with decreased pro-inflammatory cytokine and increased neurotrophic factor expression) could be obtained by Nurr1 + Foxa2-AAV injection (Supplementary Fig S6). It is well known that marker expression varies in glial cells and often changes in response to altered environments (for reviews, see Allaman *et al,* 2011; Sofroniew, 2009). Thus, it has been suggested that the detection of glial cells by immunostaining with specific antibodies is problematic (Emsley & Macklis, 2006). In fact, a proportion of AAV-infected GFP^+^ cells (26%) remained unidentified by our specific immunostaining procedure (TH^−^, GFAP^−^, Iba-1^−^). Thus, it is quite possible that more glial cells expressed exogenous Nurr1 + Foxa2 than we detected by GFAP and Iba-1 immunostaining, and that the Nurr1 + Foxa2 transgenes in the identified glia, together with the unidentified glia, were responsible for generating a significant neuroprotective environment.

Several further issues need to be addressed for the Nurr1 + Foxa2 strategy to have real clinical value. First, the forced Nurr1 + Foxa2 effects in this study were observed after imposing a *supra*-physiological dose of H_2_O_2_ treatment *in vitro* and in an artificial MPTP-PD mouse model. Thus, the therapeutic effects need to be further evaluated in PD model systems that generate physiologic levels of oxidants, and in an *in vivo* PD model that replicates better the pathologies of PD, such as the genetic PD model mouse mimicking human α-synucleinopathy. Second, constitutive long-term overexpression of Nurr1/Foxa2 may cause non-physiological perturbations of dopaminergic function. Thus, it is worth noting that, in contrast to the study of Saijo *et al* (2009) that demonstrated LPS-induced Nurr1 expression in glial cells of the adult midbrain, we were unable to detect any GFAP^+^ astrocytes and Iba-1^+^ microglia expressing these factors (not even Nurr1 expression) either *in vitro* or *in vivo* regardless of the presence or absence of toxic stimuli, suggesting that the forced expression of Nurr1/Foxa2 in glia is probably not physiologic. Thus, the clinical realization of combined Nurr1 + Foxa2 therapy absolutely requires a thorough study to rule out potential adverse effects caused by non-physiologic expression of Nurr1/Foxa2.

## Materials and Methods

### Cell cultures

#### mDA neuron-enriched cultures

mDA neurons expressing endogenous Nurr1 and Foxa2 were derived from short-term expanded VM-NPC cultures as described previously (Yi *et al*, 2014). NPCs from the VMs of mouse embryos (imprinting control region, ICR) at E10.5 were expanded *in vitro* for 3 days in serum-free N2 medium supplemented with the mitogens basic fibroblast growth factor (bFGF; 20 ng/ml; R&D Systems, Minneapolis, MN) and epithelial growth factor (EGF; 20 ng/ml; R&D Systems) and then induced to differentiate by withdrawing the mitogens. Cells were BrdU-pulsed by treating them with BrdU (10 μM, Sigma, St. Louis, MO) for 16 h prior to immunostaining with anti-BrdU and anti-TH antibodies. After 6–10 days, 10-20% of the total cells were mDA neurons expressing mature neuron- and midbrain-type DA neuron-specific markers (Fig1F, Supplementary Fig S1; Yi *et al*, 2014). In gain-of-function analyses for Nurr1 and Foxa2, primary cultures of DA neurons were established from mouse VM tissue. Briefly, VMs at E13-14 were triturated to single cells with trypsin–EDTA and plated on culture dishes pre-coated with poly-D-lysine (PDL; 25 μg/ml; Sigma, St. Louis, MO) in neurobasal medium (Life Technologies, Carlsbad, CA) supplemented with B27 and l-glutamine (Life Technologies). Ara-C (10 μM; Sigma) was added for days 3–5 to eliminate proliferating glial cells.

#### Glial cultures

Primary cultures for mixed astrocytes and microglia were derived from the VMs of mice (ICR) pups on postnatal day 1, using the protocol previously described (Saura, 2007). In brief, VMs were removed, triturated in Dulbecco's modified Eagle's medium (DMEM; Life Technologies) containing 10% fetal bovine serum (FBS; HyClone, Logan, UT), and plated in 75-cm^2^ T-flasks. When cell confluence reached 80–90%, the glia were harvested with 0.1% trypsin and prepared for use by plating on PDL-coated culture surfaces. Pure astrocytes were isolated from mouse VMs on postnatal day 5–7 and cultured in astro-medium (Heinrich *et al*, 2011). After removing microglia by gentle shaking, cells were harvested and replated in PDL-coated dishes. BV2 microglia were cultured in DMEM with 10% FBS (Blasi *et al*, 1990).

#### Mixed mDA neuron–glia culture

mDA neurons primarily cultured from E13–14 mouse VMs (total cells, 8 × 10^4^ cells) were plated on astrocyte–microglial beds (2 × 10^4^ cells/well in 24-well plates) and cultured in B27 + l-glutamine-containing neurobasal medium (Life Technologies).

### Virus production

Lentiviral vectors expressing Nurr1 or Foxa2 under the control of the CMV promoter were generated by inserting the respective cDNA into the multi-cloning site of pCDH (System Biosciences, Mountain View, CA). pGIPZ-shNurr1 and pGIPZ-shFoxa2 lentiviral vectors were purchased from Open Biosystems (Rockford, IL). The empty backbone vectors (pCDH or pGIPZ) were used as negative controls. The lentiviruses were produced and used for transducing *in vitro* cultures as described (Yi *et al*, 2014). Titers of the lentiviruses were determined using a QuickTiter™ HIV Lentivirus Quantitation kit (Cell Biolabs, San Diego, CA), and 2 ml/6-cm dish or 200 μl/well (24-well plates) with 10^6^ transducing unit (TU)/ml (60–70 ng/ml) were used for each transduction reaction. For inducing *in vivo* expression by stereotaxic injection, AAVs expressing Nurr1 or Foxa2 [tagged with hemagglutinin (HA)] under the control of the CMV promoter were generated by subcloning the respective cDNAs into pAAV-MCS vector (Addgene, Cambridge, MA). To assess the efficiency of transgene expression, GFP-expressing AAVs were also generated. Packaging and production of the AAVs (serotype 2) was performed by the Korea Institute of Science and Technology (Seoul, Korea). AAV titers were determined with a QuickTiter™ AAV Quantitation kit (Cell Biolabs). Co-expression studies were carried out by infecting cells with mixtures of the individual viral preparations (1:1, v:v).

### Preparation of glial conditioned medium

Primary glia cultures (astrocytes + microglia) expressing Nurr1 + Foxa2, Nurr1, Foxa2 alone, and empty control were prepared by lentiviral transduction. For co-expression of Nurr1 + Foxa2, lentiviruses expressing each transgene separately were mixed 1:1 (v:v) and added to cultures. Total viral volumes and titers in the cultures expressing Nurr1 or Foxa2 alone were adjusted to be same as those of the co-transduced cultures by adding control viruses. Fresh medium was added 3 days after transduction, and medium conditioned in the transduced glia was taken at 3-day interval twice. The conditioned media (CM) were filtered at 0.45 μm and stored at −80°C until use.

### Immunostaining

Cultured cells and cryosectioned brain slices were stained with the following primary antibodies: Nurr1 (1:500, rabbit, E-20, Santa Cruz Biotechnology, Dallas, TX and 1:1,000, mouse, R&D Systems); Foxa2 (1:500, goat, Santa Cruz); TH (1:250, rabbit, Pel-Freez, Rogers, AR); GFP (1:2,000, rabbit, Life Technologies); Iba-I (1:200, rabbit, Wako, Osaka, Japan); GFAP (1:200, mouse, MP Biomedicals, Santa Ana, CA); S100β (1:1,000, mouse, Sigma); TuJ1 (1:1,000, mouse, rabbit, Covance, Denver, CO); microtubule-associated protein 2 (MAP2), (1:200, mouse, Sigma); NeuN (1:200, mouse, Chemicon, Temecula, CA); DA transporter (DAT, 1:500, rabbit, Abcam, Cambridge, MA); vesicular monoamine transporter 2 (VMAT2, 1:500, rabbit, Pel-Freeze); Pitx3 (1:200, rabbit, Life Technologies); Lmx1a (1:2,000, rabbit, Millipore, Pittsburgh, PA); Nrf2 (1:200, rabbit, Santa Cruz); HA (1:200, Upstate Biotechnology, Lake Placid, NY, USA); cleaved caspase-3 (1:500, mouse, Cell Signaling Technology, Beverly, MA); and BrdU antibody(AbD Serotec, Kidlington, UK). Secondary antibodies tagged with Cy3, Cy5 (Jackson Immunoresearch Laboratories, West Grove, PA), or Alexa488 (Life Technologies) were used for visualization, and immunoreactive cells were analyzed under an epifluorescence (Leica, Heidelberg, Germany) or a confocal (Leica PCS SP5) microscope. Co-localization of Nurr1 and Foxa2 proteins in TH^+^ DA neuronal nuclei was assessed by Pearson's correlation and overlap coefficient values using the Just Another Colocalization Plugin (JACoP) of ImageJ (NIH, Bethesda, MD). In certain cases, TH-immunoreactive cells and fibers in midbrain and striatal sections were visualized by peroxidase-based colorimetric staining using a DAB substrate kit (Vector Laboratories, Burlingame, CA). Nissl staining has been done using NeuroTrace^R^ 435/455 blue fluorescent kit (Molecular Probes, Eugene, OR) based on the manufacture's protocol.

### Messenger RNA expression analysis

Total RNA preparation, cDNA synthesis, and RT–PCRs were carried out using conventional methods. Real-time PCR was performed on a CFX96™ Real-Time System using iQ™ SYBR green supermix (Bio-Rad, Hercules, CA). Gene expression values were normalized to those of GAPDH. Primers information is provided in Supplementary Table S1. High-throughput gene expression profiling for oxidative stress genes was done using a mouse oxidative stress PCR array (cat. 330231 PAMM-065ZA) using an RT^2^ Profiler PCR Array^R^ (Qiagen, Gaithersburg, MD).

### Immunoprecipitation (IP) and Western blot (WB) analysis

Interaction between Nurr1 and Foxa2 (in mouse VM tissue at 10 weeks of age) was assessed by IP. Tissues were lysed in IP lysis buffer (Thermo Scientific, Waltham, MA) supplemented with protease inhibitors. Lysates were incubated with anti-Nurr1 (1:1,000, mouse, R&D Systems) or anti-Foxa2 (1:1,000, goat, Santa Cruz) for 18–24 h at 4°C. The mixtures were shaken with magnetic beads (Life Technologies) for 1–2 h at room temperature. After washing, immunoprecipitated proteins were eluted in sample buffer and subjected to Western blot analysis with anti-Foxa2 (1:1,000, goat, Cell Signaling) or anti-Nurr1 (1:500, mouse, R&D Systems).

### *In situ* Proximity Ligation Assay (PLA)

Nurr1/Foxa2 protein interaction in the VM was further analyzed by PLA. Frozen slices of mouse VM tissues (10 weeks old) were treated with primary anti-Nurr1 (1:1,000, mouse, R&D Systems) and Foxa2 antibodies (1:1,000, goat, Cell Signaling), followed by secondary antibody, ligation, polymerization, and detection using an *in situ* PLA assay kit (Olink Bioscience, Uppsala, Sweden), as described previously (Yi *et al*, 2014).

### Measurement of ROS, NO, and arginase activity

For measurement of intracellular ROS levels, cells were incubated with 10 μM of 5-(and-6)-chloromethyl-2′,7′-dichlorodihydro-fluorescein diacetate [CM-H_2_DCF-DA (herein referred to as DCF) (Life Technologies)] for 10 min. The cells were then washed with D-PBS (in mM: 2.68 KCl, 1.47 KH_2_PO_4_, 136.89 NaCl, and 8.1 Na_2_HPO_4_), and fluorescence and phase-contrast images were taken using an Olympus (IX71, Hicksville, NY). The amount of nitrite formed from NO was measured by mixing the culture medium (50 μl) with an equal volume of Griess reagent (0.1% naphthylethylene diamine, 1% sulfanilamide, and 2.5% H_3_PO_4_). Optical density was measured at 540 nm. Arginase activity was measured with a QuantiChrom Arginase Assay kit (Bioassay Systems, Hayward, CA) according to the manufacturer's instructions.

### Animal care and experiments

All procedures for animal experiments were approved by the Institutional Animal Care and Use Committee (IACUC) at Hanyang College of Medicine under the approval number 2013-0153A. Animals were housed in a specific pathogen-free barrier facility with a 12-h light/dark cycle and maintained on standard chow (5053 PicoLab^R^ Rodent Diet 20). Animal sizes for our experiments were determined according to our *in vitro* assays and a pilot test without previous statistical calculation. Experiments were performed in accordance with the NIH guidelines. To minimize bias, behavioral assays have mostly been assessed by two experimenters in a blinded fashion.

### Stereotaxic AAV injection into MPTP-treated PD mice

Male mice (10–14 weeks old, ICR) received i.p. injections of MPTP (20 mg/kg) once daily for five consecutive days. Three days prior to, or 2 days after, the first MPTP injection, mixtures of Nurr1-AAV + control-AAV (1 μl each, 10^9^ particles/μl, for the Nurr1 group), Foxa2-AAV + control-AAV (Foxa2 group), Nurr1-AAV + Foxa2-AAV (Nurr1 + Foxa2 group), or 2 μl of control-AAV (control group) were injected over a 3-min period into ventral midbrains (3.3 mm posterior to bregma; ± 1.2 mm lateral to midline; −4.6 mm ventral to dura) under anesthesia induced by Zoletil50 (0.1 mg/kg) mixed with Rompum (93.28 μg/kg). The needle (26 gauge) was left in the injection site for 5–10 min after completion of each injection, and removed slowly. When inaccurate injection at the SN position was confirmed, the mice were excluded from analysis.

**The paper explained**

**Problem**

Parkinson's disease (PD) is a common movement disorder characterized by progressive degeneration of dopamine (DA)-secreting neurons (DA neurons) in the midbrain. No restorative and disease-modifying therapies are currently available for PD. Use of the physiological mechanisms promoting midbrain DA (mDA) neuron survival seems an appropriate option for developing treatments for PD.

**Results**

We showed that Nurr1 and Foxa2, the transcription factors expressed in midbrain DA (mDA) neurons, physiologically interact for the survival of these neurons. However, their expressions decline during aging and degenerative processes. In addition to cell-autonomous actions in mDA neurons, Nurr1 + Foxa2 transgene expression in glial cells creates a neuroprotective environment by (i) suppressing the secretion of pro-inflammatory cytokines as well as (ii) promoting neurotrophic factor release. As a consequence of these bimodal actions, adeno-associated virus (AAV)-mediated gene delivery of Nurr1 and Foxa2 in a PD mouse model markedly protected mDA neurons and motor deficits associated with nigrostriatal DA neurotransmission in PD.

**Impact**

Based on our observations, we propose that combined Nurr1 + Foxa2 gene delivery can be a promising therapeutic tool in PD.

### Histological measurement of TH-immunoreactive cells

Four weeks to 1 year after AAV injection, animals were anesthetized and perfused intracardially with 4% paraformaldehyde in PBS. Brains were removed and immersed in 30% sucrose in PBS overnight and sliced on a freezing microtome (CM 1850; Leica, Wetzlar, Germany). Midbrain and striatal sections (30 μm thick) were subjected to TH immunohistochemistry as described above. The total number of mDA neurons in the right and left sides of the midbrain was obtained by counting TH-immunoreactive cells throughout the midbrain (a total of 11–14 sections counted for each animal). The Abercrombie correction factor [*N *= *n *× *T*/(*T *+ *D*)], where *N* is the actual number of cells, *n* is the number of nuclear profiles, *T* is the section thickness (30 μm), and *D* is the average diameter of nuclei, was used to compensate for double counting in adjacent sections.

### Behavior tests

#### The apomorphine-induced rotation test

Apomorphine (Sigma) was subcutaneously injected at a dose of 0.5 mg/kg and rotation was monitored for 60 min. The results were expressed as net turns/60 min.

#### The cylinder test

Mice were placed in a small transparent cylinder (height, 15.5 cm; diameter, 12.7 cm), and the numbers of right and left forelimb contacts with the wall of the arena while rearing were recorded for 3 min.

#### The pole test

Animals were placed head upwards on top of a vertical wooden pole 50 cm in length (diameter, 1 cm). The base of the pole was placed in the home cage. Once placed on the pole, animals oriented themselves downward and descended the length of the pole back into their home cage. All of the animals received 2 days of training that consisted of five trials for each session. On the test day, animals received five trials, and the time to orient downward was measured.

#### The challenging beam traversal test

Motor performance was measured with a novel beam test adapted from traditional beam-walking tests. Briefly, the beam (length, 1 m) started at a width of 3.5 cm and gradually narrowed to 0.5 cm in 1-cm increments. Animals were trained to traverse the length of the beam, starting at the widest section and ending at the narrowest section for 2 days before real testing. Times required for the animals to traverse the beam were measured.

#### The locomotor test

A 40-cm square cage with a peripheral and a central area was used for the locomotion test. Mice were placed in the center of the open field and allowed to freely explore the apparatus for 20 min while being tracked by a video-recording system. After the test, each mouse was returned to its home cage and the open field was cleaned with 70% ethyl alcohol and permitted to dry between tests. To assess the process of habituation to the novelty of the arena, mice were exposed to the apparatus for 20 min on two consecutive days for statistical analysis.

### Cell counting and statistical analysis

Immunostained and DAPI-stained cells were counted in 10–20 random areas of each culture coverslip using an eyepiece grid at a magnification of 200× or 400×. Data are expressed as the mean ± SEM of three to ten independent cultures. Normal distribution of the data in this study was confirmed by Kolmogorov–Smirnov test. For every figure, statistical tests are justified as appropriate. Statistical comparisons were made using Student's *t*-test (unpaired or paired) or one-way ANOVA followed by Bonferroni *post hoc* analysis using SPSS® (Statistics 21; IBM Inc.). The *n*, *P*-values, and statistical analysis methods are indicated in the figure legends.

## Supporting Information

Supplementary Figures S1–S7, Supplementary Table S1

Review Process File
