# Supplementary material for: Combined Nurr1 and Foxa2 roles in the therapy of Parkinson's disease
Source: EMBO Mol Med. 2015 Mar 10;7(5):510–25. doi: 10.15252/emmm.201404610 (PMC4492814; doi:10.15252/emmm.201404610)

**Supplementary Information**

**Contents**

| Supplementary Figure 1.  Midbrain-type DA neurons derived by in vitro differentiation of VM-NPCs …………………………………………………………........................................................ | 2 |
| --- | --- |
| Supplementary Figure 2.  Expression of antioxidant genes in mDA cultures pre-treated with N-CM, F-CM or NF-CM and analyzed by RT^2^ PCR Profiler Array^R^ ……………................................................. | 4 |
| Supplementary Figure 3.  AAV-mediated transgene expression in mDA neurons, astrocytes , and microglia in the SN of mouse midbrains ………………................................................................................. | 6 |
| Supplementary Figure 4.  Comparison of midbrain TH+ mDA neuron numbers and striatal TH+ fiber intensities…………………………………............................................................................ | 7 |
| Supplementary Figure 5.  Neuroprotective effect by Nurr1+Foxa2-AAV injection is further confrmed by Nissl and NeuN staining ………………………………........................................................................ | 9 |
| Supplementary Figure 6.  Comparison of S100β, RAGE, GFAP, pro-inflammatory cytokines and neurotrophic factors expression in C-AAV- and NF-AAV-injected VMs of MPTP-treated PD mice………………………………........................................................................................ | 11 |
| Supplementary Figure 7.  Up-regulation of jmjd3 mRNA expression in BV2 microglia by forced Nurr1 and Foxa2 expression………………………………...............................................................................  Supplementary Table 1.  Primer sequences used for qPCR reactions……………….................................................... | 12  13 |

**
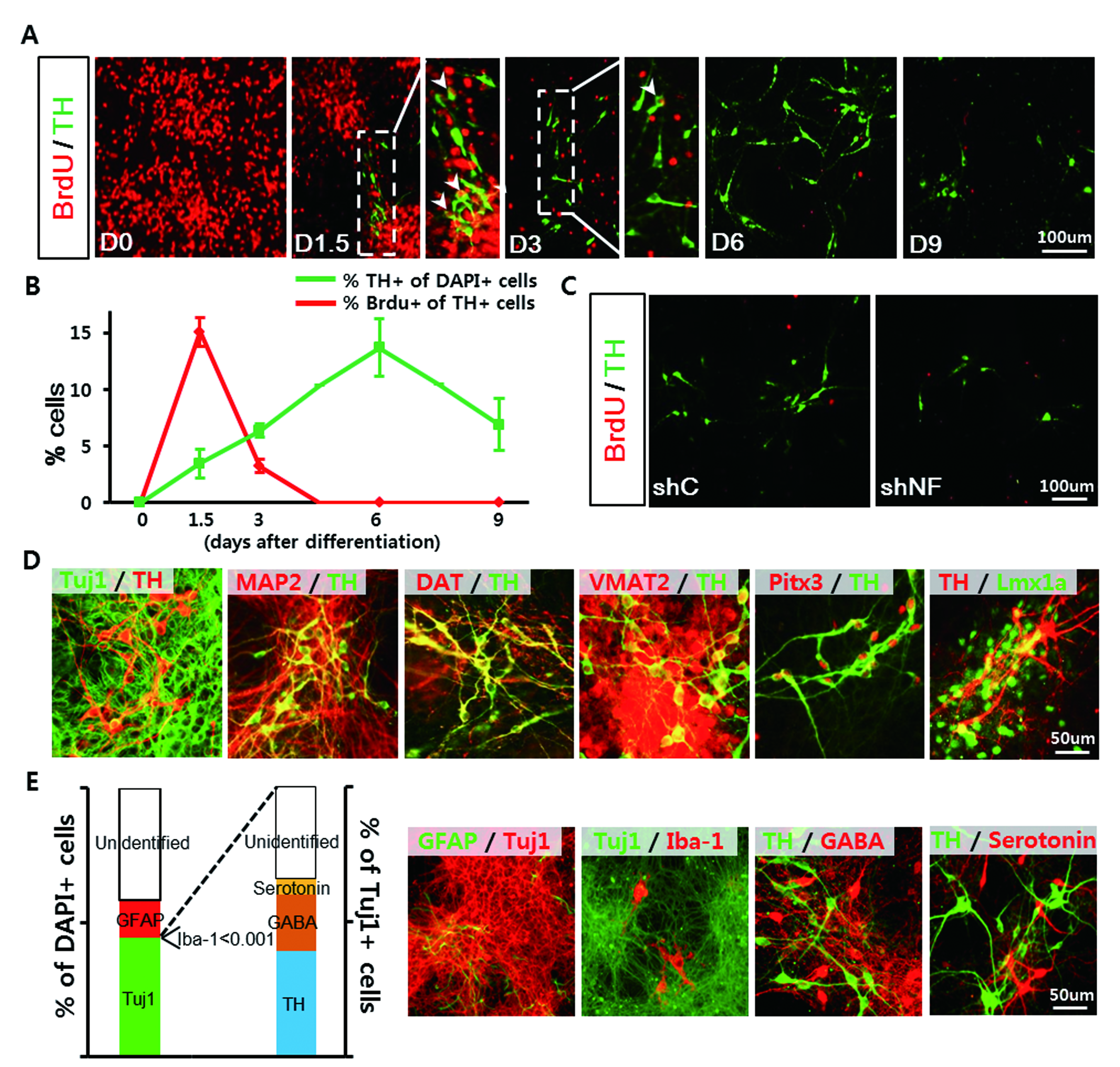
**

**Supplementary Fig S1**. Midbrain-type DA neurons derived by in vitro differentiation of VM-NPCs. NSCs derived from mouse embryonic VMs at E10.5 were expanded for 3-4 days in the presence of bFGF+EGF, and induced to differentiate by withdrawal of the mitogens. A-C, TH+DA neuron production during VM-NSC differentiation. TH+ cells are gradually generated and reach a maximum 6 days after differentiation (A,B). In a BrdU pulse experiment, the maximum number of BrdU+/TH+ cells (newborn DA neurons) is present on differentiation day 1.5 (D1.5). TH+/BrdU+ cells (indicated by arrowheads) in the boxed areas at D1.5 and D3 are enlarged in both right flanks. Note that none are BrdU+/TH+ at D6-D9 in a Nurr1 and Foxa2-knockdown experiment of the kind shown in Fig. 1. (A,B), regardless of the shRNA treatment (C). D, The midbrain DA neuronal phenotypes of the TH+ cells at D9 are confirmed by co-staining against markers specific for neurons (TuJ1, MAP2), DA homeostasis (DAT, VMAT2), and midbrain-type DA neurons (Pitx3, Lmx1a). D, The other cell types present in the differentiated VM-NSC cultures.


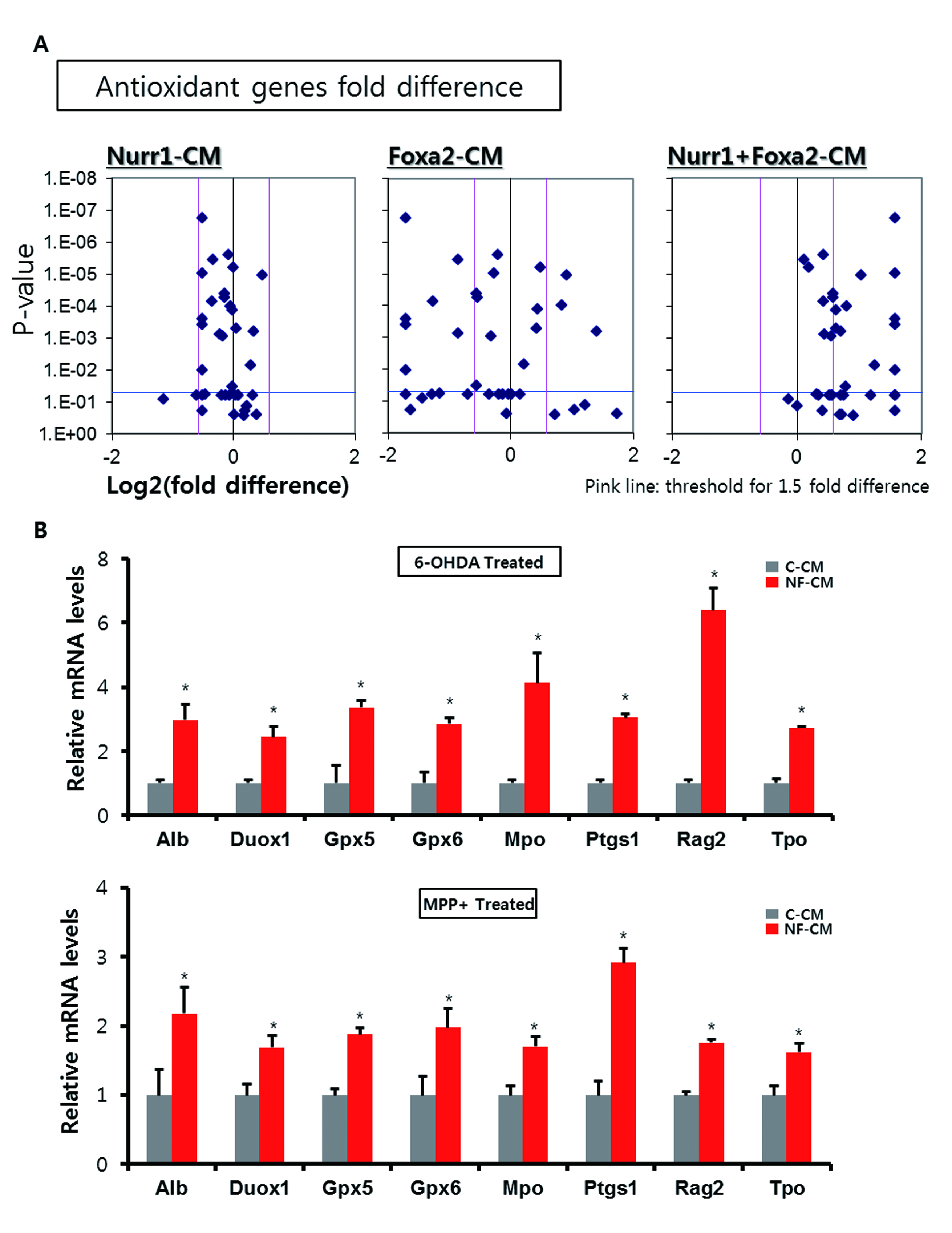


**Supplementary Fig. S2.** A, Expression of antioxidant genes in mDA cultures pre-treated with N-CM, F-CM or NF-CM and analyzed by RT^2^ PCR Profiler Array^R^. Log2-fold differences of gene expressions, compared with C-CM treated cultures, are shown by Volcano plots. B, The NF-CM treatment effect on the anti-oxidant gene expression was further confirmed in MPP+ (250 uM) and 6-OHDA (500 uM)-treated cultures. Top-ranked 8 anti-oxidant genes in Fig. 3F were selected and their expressions were tested using real-time PCR. Data represent means±SEM of 3 PCR reactions.*Significantly different from the control (C-CM) at *P*<0.05, n=5 culture wells in each group. *P*-values: 0.038 (Alb), 0.026 (Duox1), 0.013 (Gpx5), 0.036 (Gpx6), 0.042 (Mpo), 0.038 (Ptgs1), 0.026 (Rag2), and 0.013 (Tpo) for the 6-OHDA treated values, 0.024 (Alb), 0.037 (Duox1), 0.033 (Gpx5), 0.031 (Gpx6), 0.044 (Mpo), 0.011 (Ptgs1), 0.029 (Rag2), and 0.035 (Tpo) for the MPP+-treated values, unpaired student's *t*-test.

**
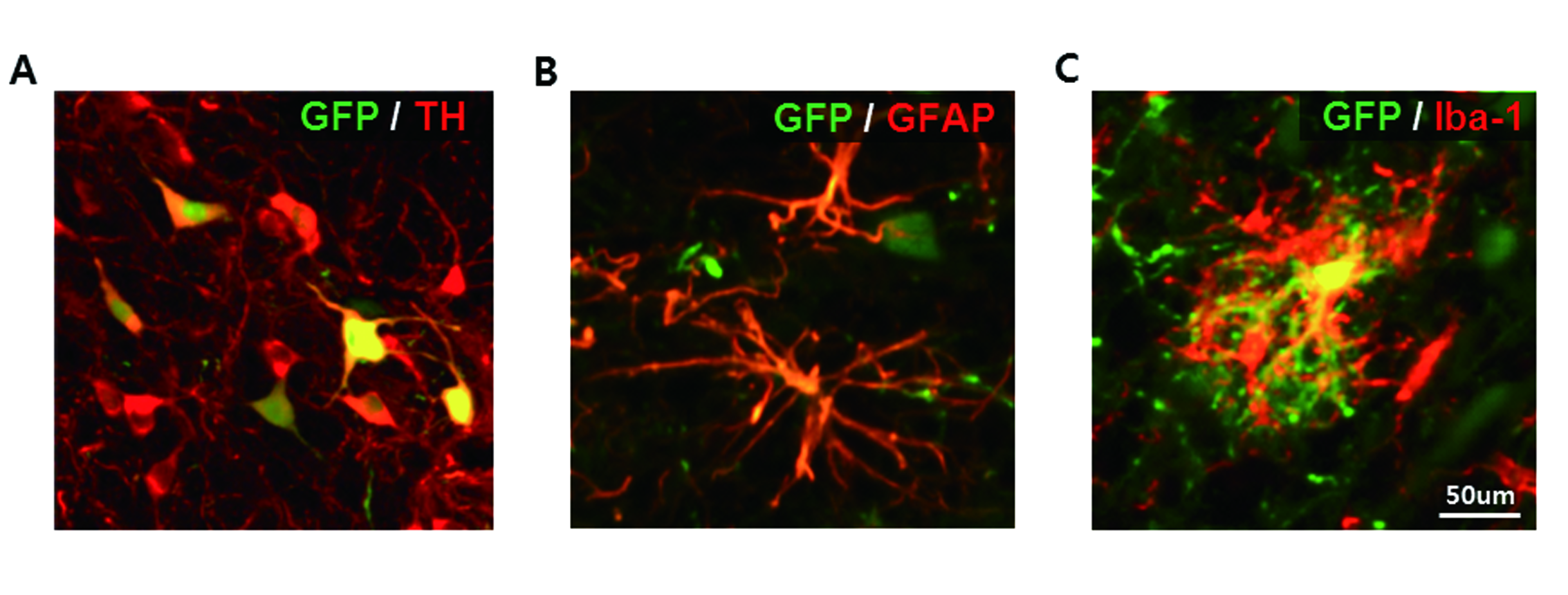
Supplementary Fig. S3.** AAV-mediated transgene expression in mDA neurons (A), astrocytes (B), and microglia (C) in the SN of mouse midbrains. GFP-expressing AAVs were injected into the SN of mouse midbrains (10 weeks old). One week later, the SN was cryosectioned and double immunostained for GFP/TH, GFP/GFAP, and GFP/Iba-1 to detect co-localization of exogenous GFP expression in mDA neurons, astrocytes, and microglia, respectively.


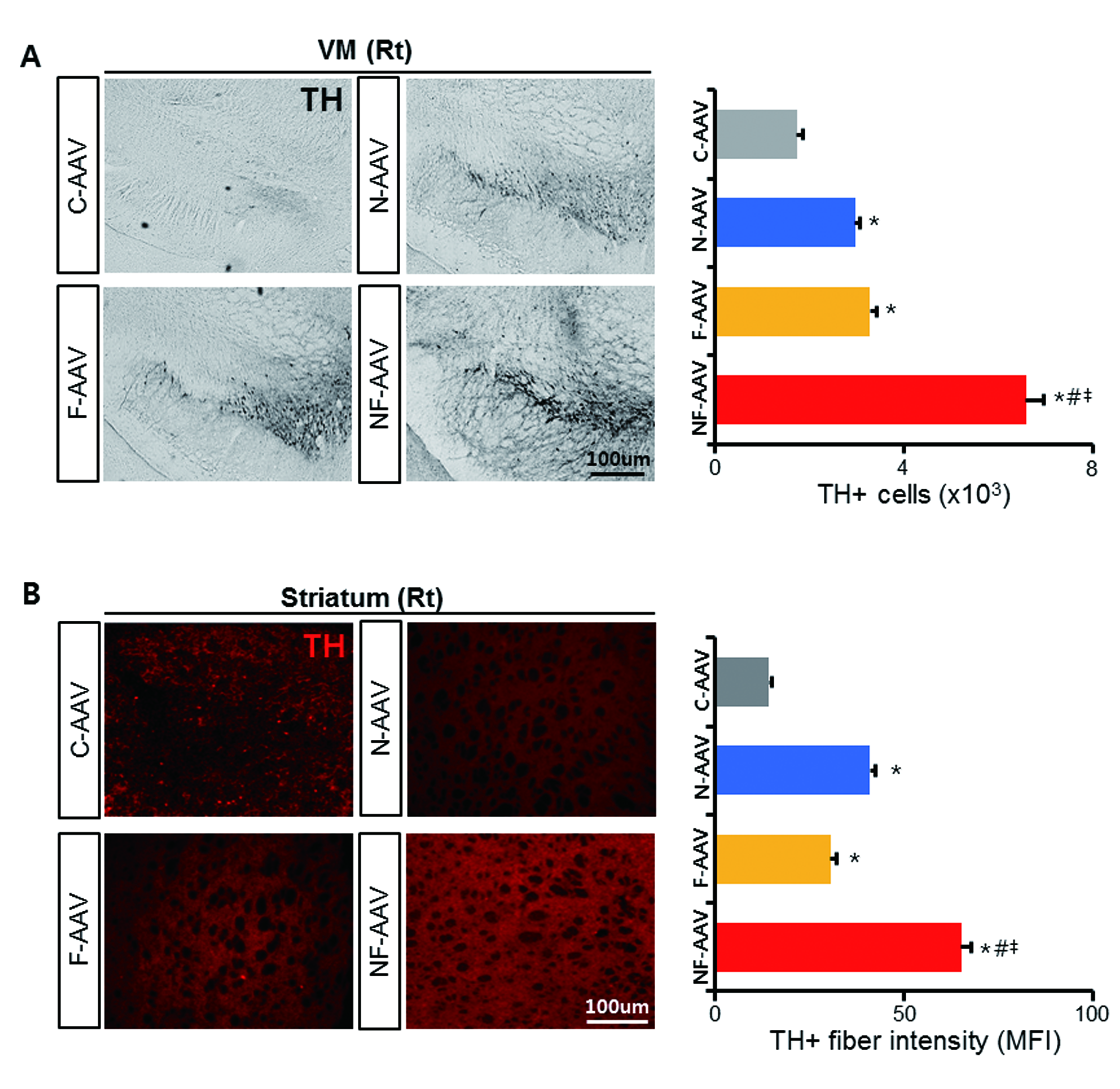


**Supplementary Fig. S4.** Comparison of midbrain TH+ mDA neuron numbers (A) and striatal TH+ fiber intensities (B) on right sides injected with C-, N-, F-, and NF-AAVs. The AAVs were injected into the right sides of midbrains 3 days prior to the initial MPTP treatment. TH+ cell numbers in the midbrain, and TH+ fiber intensities in the striatum, were measured 8 weeks later. After staining, TH immunoreactive intensities (MFI) of 50 microscopic fields in each side (from 7-10 striatal sections) per animal were measured. Shown in the graph of B are the MFI values of the right relative to the left striatum. Significantly different from C-AAV*, N-AAV#, F-AAV‡ at p<0.05, n=12 mice, *P*-values: 0.015 (N-AAV*), 0.011 (F-AAV*), 0.007 (NF-AAV*), 0.041 (NF-AAV#), and 0.022 (NF-AAV‡) for the TH+ cell counts, 0.012 (N-AAV*), 0.029 (F-AAV*), 0.011 (NF-AAV*), 0.034 (NF-AAV #), and 0.018 (NF-AAV ‡) for the MFI values, One-way ANOVA followed by Bonferroni *post hoc* test.

**
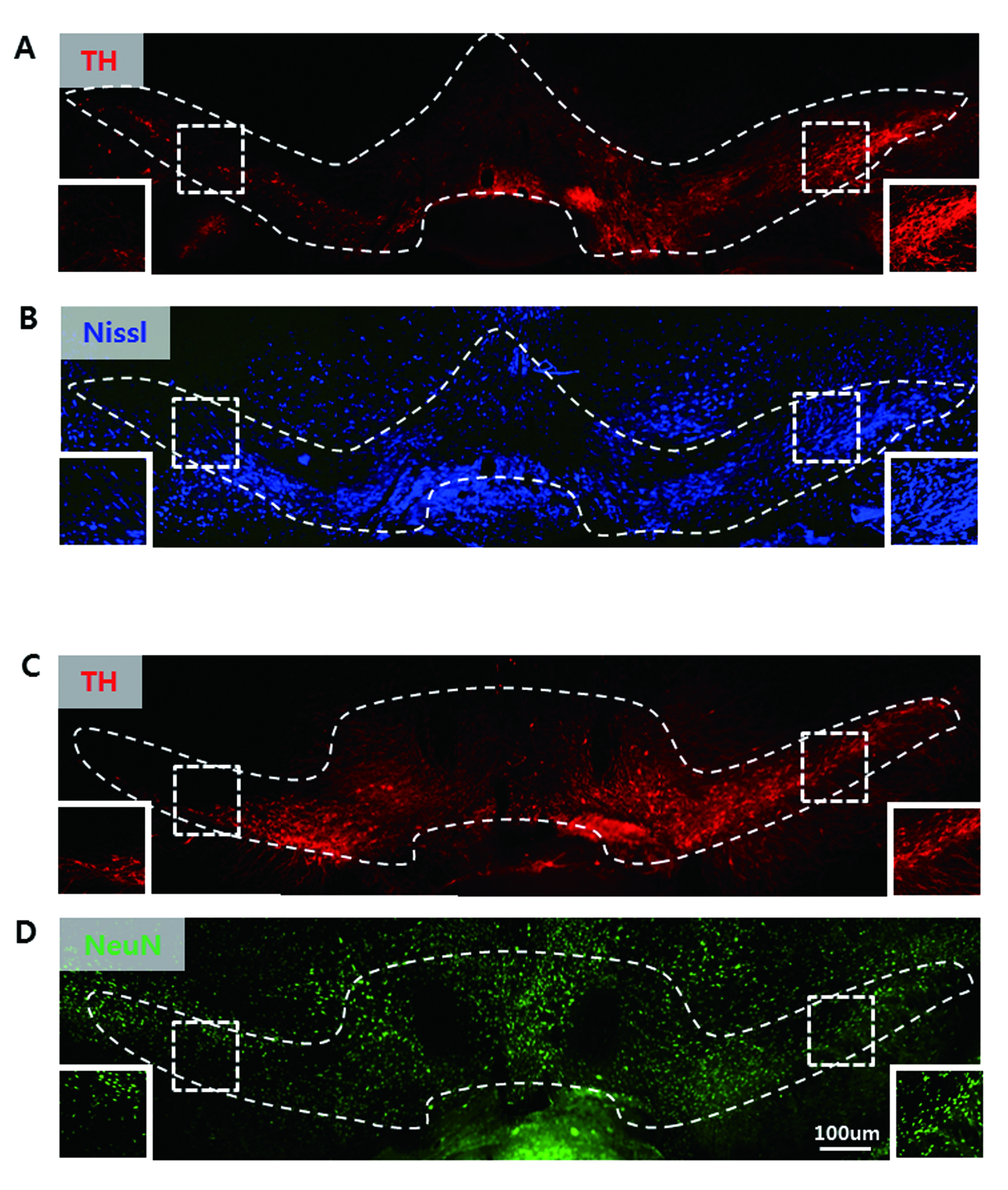
**

**Supplementary Fig. S5.** Neuroprotective effect by Nurr1+Foxa2-AAV injection is further confrmed by Nissl and NeuN staining. As described in the legend of Fig. 5, Nurr1+Foxa2-AAV and control-AAV was injected on right and left sides of PD mouse, respectively. Four weeks later, midbrain sections were double stained with TH/Nissl and TH/NeuN. Nissl staining has been done using NeuroTrace^R^ 435/455 blue fluorescent kit. The images of TH (A) and Nissl (B) were taken from an identical section, while those of TH(C) and NeuN (D) were from another midbrain section. SN and VTA regions in each image are outlined with dotted line. Symmetric right and left SN regions (indicated with boxes) are enlarged in insets. It is noted that, similar to TH+ cells, the numbers of Nissl- and NeuN-stained neurons are greater in Nurr1+Foxa2-AAV injected sides than those of the control.

**
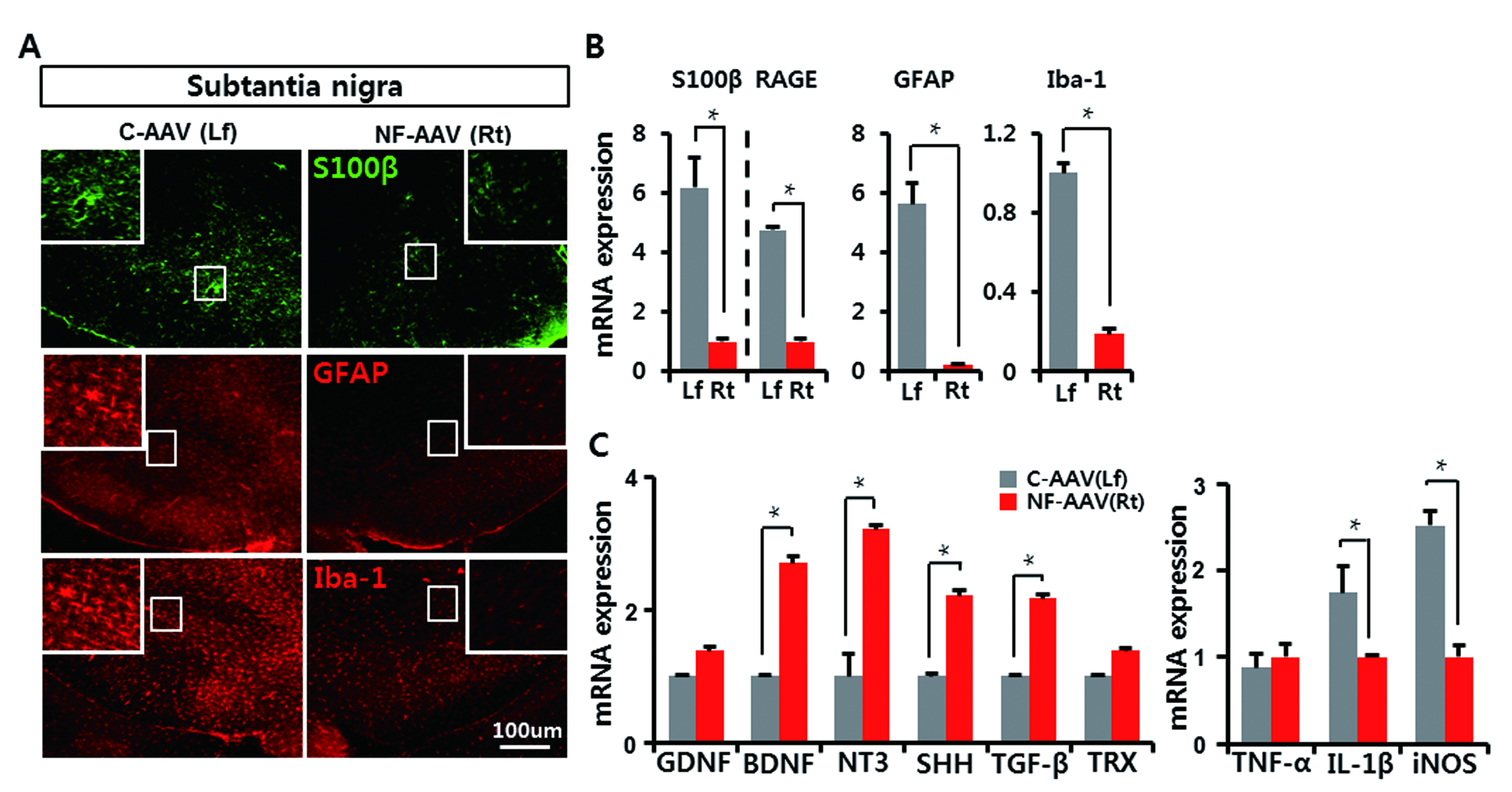
**

**Supplementary Fig. S6.** A-B, Comparison of S100β, RAGE and GFAP expression in C-AAV- (left) and NF-AAV-injected (right) VMs of MPTP-treated PD mice. The AAVs were injected 3 days prior to the initial MPTP treatment. Expression was assessed by immunohistochemical staining (A) and real-time PCR (B) analyses 2 weeks after AAV injection. Insets in A are high-power images of the boxed areas. Messenger RNA levels of the pro-inflammatory cytokines and neurotrophic factors indicated were also compared between the right and left sides of midbrains (C). *Significantly different at p<0.01, n= 3 PCR reactions each, *P*-values: 0.000 (S100β), 0.001 (RAGE), 0.000 (GFAP), 0.000 (Iba-1), 0.000 (BDNF), 0.000 (NT3), 0.001 (SHH), 0.000 (TGF-β), 0.000 (IL-1β), and 0.000 (iNOS), paired student's *t*-test.


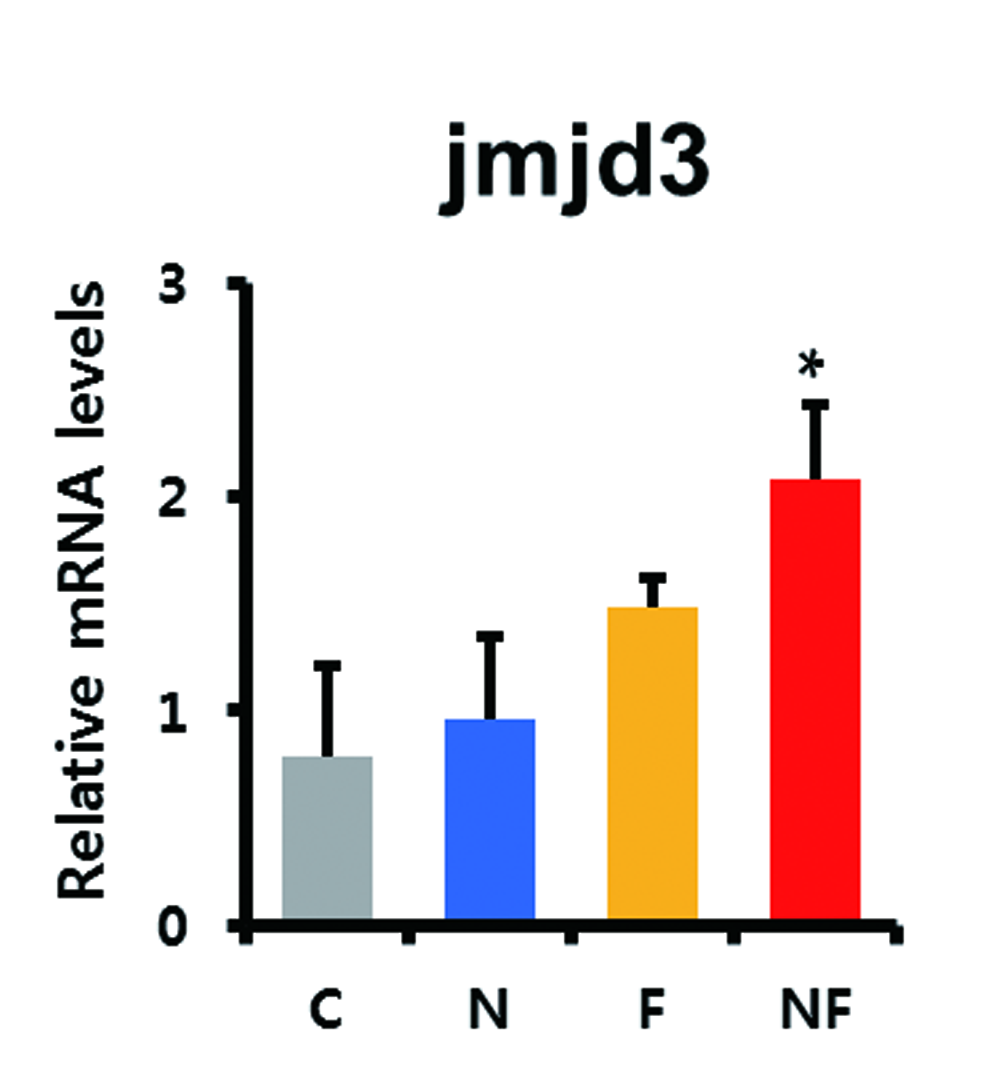


**Supplementary Fig. S7.** Up-regulation of jmjd3 mRNA expression in BV2 microglia by forced Nurr1 and Foxa2 expression. *Significantly different at *P=*0.023, n= 3 PCR reactions each, One-way ANOVA followed by Bonferroni *post hoc* test.

**Supplementary Table 1. Primer sequences used for qPCR reactions.**


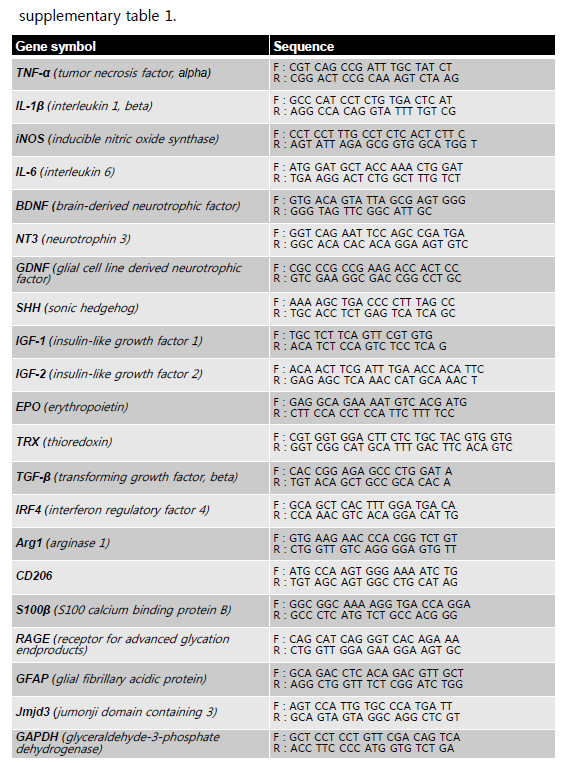

Supplement: Supplementary file 1 [file emmm0007-0510-sd1.docx]
