# Supplementary material for: Combined Nurr1 and Foxa2 roles in the therapy of Parkinson's disease
Source: EMBO Mol Med. 2015 Mar 10;7(5):510–25. doi: 10.15252/emmm.201404610 (PMC4492814; doi:10.15252/emmm.201404610)
Supplement: Supplementary file 2 [file emmm0007-0510-sd2.pdf]

EMBO Molecular Medicine

Combined Nurr1 and Foxa2 roles in the therapy of Parkinson's disease

Sang-Min Oh, Mi-Yoon Chang, Jae-Jin Song, Yong-Hee Rhee, Eun-Hye Joe, Hyun-Seob Lee, Sang-Hoon Yi and Sang-Hun Lee

Corresponding author: Sang-Hun Lee, College of Medicine

Review timeline:

Submission date:	05 September 2014
Editorial Decision:	30 September 2014
Revision received:	29 December 2014
Editorial Decision:	26 January 2015
Revision received:	10 February 2015
Accepted:	12 February 2015

Transaction Report:

(Note: With the exception of the correction of typographical or spelling errors that could be a source of ambiguity, letters and reports are not edited. The original formatting of letters and referee reports may not be reflected in this compilation.)

Editor: Céline Carret

1st Editorial Decision 30 September 2014

Thank you for the submission of your manuscript to EMBO Molecular Medicine. We have now heard back from the three referees whom we asked to evaluate your manuscript. Although the referees find the study to be of potential interest, they also raise a number of concerns that need to be carefully and rigorously addressed in the next version of your article.

As you will see from the reports pasted below, all three referees find the study important and novel. However, the referees question the relevance of the gene therapy approach in terms of longevity and translational potentials. They also mention a number of technical limitations that have to be experimentally addressed when appropriate. Finally, they all suggest ways to improve conclusiveness and clarity and we would strongly encourage you to address all concerns as best as you can.

Should you be able to address these criticisms in full, we would be willing to consider a revised manuscript. However, please note that that it is our journal's policy to allow only a single round of revision, and that acceptance or rejection of the manuscript will therefore depend on the completeness of your response and the satisfaction of the referees with it.

EMBO Molecular Medicine has a "scooping protection" policy, whereby similar findings that are published by others during review or revision are not a criterion for rejection. Should you decide to submit a revised version, I do ask that you get in touch after three months if you have not completed it, to update us on the status.

Please also contact us as soon as possible if similar work is published elsewhere. If other work is published we may not be able to extend the revision period beyond three months.

I look forward to receiving your revised manuscript.

***** Reviewer's comments *****

Referee #1 (Comments on Novelty/Model System):

The neuroprotective role of Nurr1 and Foxa2 has not been previously explored, in particular via the action of these transcription factors in glial cells.
The main weaknesses of the work is that AAV-mediated gene delivery does not infect glial cells at high efficiency and therefore it is unclear if the in vivo experiment demonstrates neuroprotection via the action of Nurr1 and Foxa2 in glial cells, as suggested by the authors.
In addition, the therapeutic efficacy of this approach remains uncertain as the MPTP model does not fully replicate the pathogenesis of Parkinson's disease. Complementing this study with genetic models of PD would be useful, but I understand that this implies a new set of experiments that could be difficult to achieve in a reasonable time frame. It would be for instance interesting to determine if Foxa2 and/or Nurr1 knockdown leads to increased vulnerability to alpha-synuclein.
The authors suggest that gene therapy should be envisaged to overexpress these transcription factors. However, some caution is needed as long-term overexpression of transcription factors could lead to deleterious effects that make this approach unlikely to be approved as such. In addition, it is unclear if glial cells actually express Foxa2 and Nurr1 in physiological or diseased conditions, and therefore unexpected long-term effects have to be expected following forced expression in these cell types.

Referee #1 (Remarks):

This article by Oh and colleagues reports on the neuroprotective effects of the combined expression of Nurr1 and Foxa2 on mouse dopaminergic neurons in vitro and in the MPTP model of Parkinson's disease. They identify an unexpected mode of action of these transcription factors via neighboring glial cells, and highlight the cooperation between Nurr1 and Foxa2 in neuroprotection. However, it is unclear if Foxa2 and Nurr1 are expressed in glial cells in physiological conditions, possibly in response to oxidative stress for instance, or if the effects observed are only the results of forced expression in these cells. In this case, it would raise some concerns regarding the long-term consequences of transgenic expression of these transcription factors for therapeutic purposes.
This is a thorough study, well executed and the manuscript is well written.

The following major comments should be addressed before publication:

Fig. 1D: the blot showing co-immunoprecipitation of Foxa2 is not convincing. There is no indication of molecular weight in the western blot and the Foxa2 signal is very weak.

Fig. 1F: what are the cells that are TH-negative? Is there evidence for expression of Foxa2 and Nurr1 in non-neuronal cells?

Fig. 1G: the authors should provide evidence for the efficacy of the shRNA against Foxa2 and Nurr1. Knockdown could be assessed by immunocytochemistry with specific antibodies.
Also, it is unclear if the number of TH+ cells is decreased in shN, shF and shNF in the absence of any exposure to H_2O_2.

Most of the in vitro experiments using H_2O_2 as a stressor find significant effects of changes in Foxa2 and Nurr1 expression at H_2O_2 doses of 500µM. This is a non-physiological dose of H_2O_2 that may engage processes that are very different from the in vivo situation. This point should be at least discussed.

Fig. 5: as the forced expression of Foxa2/Nurr1 is expected to induce expression of dopaminergic

markers, the number of Nissl-positive neurons should be quantified. This would help to determine what is the contribution of de novo expression of dopaminergic markers in the substantia nigra and determine the exact extent of the protection of resident neuronal cells.

The authors do not seem to use unbiased stereology for the assessment of the number of neurons in the substantia nigra, although stereology is now considered as the method of choice in the field. It is unclear what is the number of sections counted in this study. This should be better reported in the Material and Methods section.

It is important to determine the in vivo effects of the forced long-term expression of Foxa2 and Nurr1 in the substantia nigra in the absence of any MPTP-induced lesion. This would determine the possible side effects of such a gene therapy approach. Indeed, constitutive long-term overexpression of these transcription factors is likely to cause non-physiological perturbations of the dopaminergic function.

Supplementary figure S3: the tropism of the AAV vector used in this study should be better reported, if possible with a quantitation of the neuronal vs glial transduction. It is stated in the discussion that AAV transduces less than 5% of glial cells. It would be useful to determine the percentage of GFP-positive cells that are TH+, GFAP+ and Iba1+. This is important as the authors suggest that some of the observed neuroprotective effects are due to changes in the glial rather than neuronal cells. If less than 5% of the transduced cells are glial cells, it is unlikely that glial cells may play a prominent role in the observed in vivo results.

The genes Foxa1 and Foxa2 have been reported to have redundant functions. In the Foxa2 knockdown experiments, what is the status of Foxa1?

Minor comments:

Fig. 3B: the description of the statistics on the graph is not very clear.

Fig. 5D: in contrast to what is stated in the Result section, apomorphine is indicated to induce "contralateral" rotations. If this means contralateral to the AAV-injected side, it appears unlikely to be the case.

Fig. 5E: please clarify how asymmetry in the cylinder test is expressed.

It is unclear how the vector titer was determined. Did the author use the number of particles containing a genome as determined by real-time PCR?
In the group injected with the Foxa2 and Nurr1 vectors, did the authors double the injected dose of AAV vector as compared with the groups injected with individual vectors? This should be more accurately stated in the Material and Methods section.

What is the promoter used to express Foxa2 and Nurr1 with AAV vectors in vivo?

Typographical mistakes:
Abstract section:
Line 9: "protects"
Line 12: "nigrostriatal"

Page 3, line 6: "nigrostriatal"

"transgene" should be used instead of "exogene" throughout the manuscript.

Page 36: "*p=0.0000... " should be corrected
Page 36, line 9: "microscopic"

Referee #2 (Remarks):

In the following manuscript, Oh and co-workers report the synergistic neuroprotective effect of Nurr1 and FOXA2 in an animal model of PD.
Their rational seeds from an observation that Nurr1 and FoxA2 transcription factor expression is lost or decreased, with age in mice; and that knock-down of both TFs dramatically decrease TH expression while increasing cell death, in vitro.
Using a battery of in vitro and in vivo tests, they demonstrate that overexpression of both TFs in DA neurons or glia is neuroprotective to DA neurons, and in case of mis-expression is associated with reduced inflammation which is a process otherwise known to exacerbate DA neuron death.
Remarkably, the authors show that overexpression of both TFs in vivo prevents DA neuron death in response to MPTP treatment.
My only main concern is that all observations made in vitro may results from ongoing neurogenesis, rather than protecting TH neurons from death, especially since embryonic cells were used. Time course studies would clarify this issue.

The manuscript otherwise reads wells; the message is simple and straightforward; the research is novel since most experiments were conducted in adult animals, although the authors use embryonic cells and cell lines to confirm or give a rational to some of their work, which in some instances may not be relevant.

Comments to authors:

Figure 1:
Panel D: Blots really need improvement
Panel G:
- Pictures are misleading: DAPI staining reveals an increase in the number of cells with shNF targeted KO (2-3 fold compare to control). Since the number of C-Capase3 positive cells also increases, the ratio of cell death seems null. Can the author comment on that?
- Data should also be presented as % of DAPI, not only TH+ per well (this is not indicative at all).
- The authors need to add images for appreciation of Nurr1 and FOXA2 downregulation following sh KO. Does shNurr1 decrease FOXA2 expression? Does shFOXA2 decrease Nurr1 expression? Show pictures.
- Are other neuronal subtypes affected by the treatment, in other words are only TH+ cells dying or could interneurons (GAD65/67) present in the culture die at the same rate, speed?
- The authors used sh vectors leading to partial KO (reduction of 30-40%), moreover, it is possible that double infection with shNurr1 and shFOXA2 viruses is toxic per se. What MOI was used in these experiments? Could the authors show that neuronal death is TH neurons-specific?
- Have the authors checked that the decrease of TH+ cells is not due to 1) lack of ongoing TH neuron genesis due to F and N KO or 2) lack of maturation of the progenitors, which undergo neuronal death, as they cannot mature (were BrdU pulses performed?). It is highly possible, since embryonic cells were used, that the shN, shF and shNF KO prevent maturation of progenitors. Is the number of TH+ cells identical for all conditions on day 1, prior to sh KO?

Figure 2:
Panel A and panel D:
- TH staining intensity for young (panel A) and "minus MPTP" should be identical, since the age of animal is identical.
- Data on decrease in intensity should be completed with TH+ quantification (preferably by stereology).
- FOXA2 intensity panel A and B should be identical since animals are of same age. If those are 2 different batches, data should be presented as % of control.
- Why is TH staining lost at 18 months of age? Dawson and Dawson (nature neuroscience 2013) show sustain staining intensity for TH in animals aged 20 months. Could this be strain specific? Why are Nurr1 and FOXA2 staining brighter in panel D (minus MPTP) compare to panel A 10 weeks young?--> those should be identical.
Panel B:
- Bands for FOXA2 missing while a decrease is mentioned in figure legend and text; please correct (also, indicate molecular weight of the bands).

© EMBO

- Is TH maintained or also decreased? Please add Western Blot data for TH.
Panel C and E:
- Add TH quantification
- Are Nurr1 and FOXA2 expression lost because TH neuron age? Counting shows a 2 fold decrease in F and N staining, but images report a complete loss of the markers: what is the proportion of TH still expressing the TFs at D24?
- Are other DA determinant decreased or lost? Lmx1a, lmx1b, En1 (this could be checked by RT-PCR or qPCR). How about neuronal markers VMAT2, AADC?

Figure 3:
- Panel D: is increase in TH due to ongoing neurogenesis? Have the authors tried to block cell proliferation to really prove maturation occurs? Have the authors exposed the cultures to BrdU and stain for BrdU /TH to rule out ongoing TH neuron genesis? One way to address neurogenesis is to present TH counts out of DAPI (absolute, per field of view), and as ratio TH/DAPI: if ratio close to one, one can conclude ongoing neurogenesis occurs, leading to increase in absolute number of TH+ cells.
- RT-qPCR for anti-oxidant in cultures treated with MPP+ and 6-OHDA are missing.

Figure 4:
- Panel A: As opposed to previous work from Saijo et al (2009, Cell), Nurr1 has a little neuroprotective effect; could the authors comment on this discrepancy?
- All panels together show that NF overexpression dramatically increases expression (probably secretion) of neurotrophines, and is associated with decrease expression of pro-inflammatory mediators. This in vitro data were unfortunately not confirmed in vivo. Therefore, the reviewer is still not sure what this figure adds to the study. Could some of the findings presented in this figure be extended to the in vivo part (e.g counting IBA1 staining (reduction should be observed when NF are injected in the SN of the animals), staining for growth factors, etc)?
- To the reviewer's knowledge, SHH is by default increased with inflammation; has this been tested (LPS treated vs non-LPS treated)?

Figure 5:
- Overall comment:
* Stereological counting is mandatory when working with animal sections (panel B: TH quantification).
* High magnification images are needed to appreciate the number of TH+ neurons and their morphology.
* Why was not the effect of each single factor assessed? Perhaps, and in light with the work conducted by Decressac and co-workers, either Nurr1 or FOXA2 would have been sufficient to induce cellular and locomotor recoveries.
* Assessment of inflammation is required to give a rational to data presented in figure 4: GFAP, IBA1 quantifications are necessary here.
- Panel M: what cells are targeted with the AAV employed? Are they mainly neurons or glia? Is the effect of combined overexpression of N and F direct or indirect? If AAV used, the injections should be performed in the striatum to have a specific expression of both TFs in DA neurons.
- Panel P: assessing TH expression 14 days post-treatment and lesion is rather quick. Why not assessing TH counts at 4 and 8 weeks as well (like in panel A)?

Referee #3 (Comments on Novelty/Model System):

The authors show an impressive amount of in vitro and in vivo data to support that elevating the expression of Nurr1 and Foxa2 has a synergistic effect not only in improving PD-related symptoms but also in improving mDA neuron survival. Overall, this is a well-designed and nicely performed study with a focus of high biological and translational interest.

Referee #3 (Remarks):

This work by Oh et al reports a new way, i.e. coexpression of Nurr1+Foxa2, to improve the survival of mDA neurons in PD models. As opposed to current PD therapies, this approach has the potential of changing the disease course in addition to symptomatic improvement. In this manuscript, the authors show an impressive amount of in vitro and in vivo data to support that elevating the expression of Nurr1 and Foxa2 has a synergistic effect not only in improving PD-related symptoms but also in improving mDA neuron survival. Mostly importantly, the neuroprotective effect of Nurr1+Foxa2 is sustained after 6 months. In addition, this study also elucidates the cellular and molecular mechanisms that underlie the effect of Nurr1+Foxa2 coexpression. It shows that Nurr1+Foxa2 acts by suppressing the secretion of pro-inflammatory cytokines and increasing the secretion of neurotrophic factors in neighboring glia and by up-regulating Nrf2 and anti-oxidant genes in mDA neurons. The authors present their findings logically and clearly. Overall, this is a well-designed and nicely performed study with a focus of high biological and translational interest. I only have a few minor suggestions that need to be addressed.

1) It would be nice to add a simple diagram in the end that summarizes the cell-autonomous and paracrine activities of Nurr1+Foxa2 on VM neuron and glia.

2) Double check the registration of the boxed areas with the enlarged panels in Fig. 1E, Fig. 1G, and Fig. 2A.

3) In Fig. 1G, it is better to provide color-coded names of the gene products (TH, Casp3, DAPI) directly within the images.

4) The protein signal for Foxa2 is missing in Fig. 2B western blot.

5) Single letter abbreviations, such as N and F, should be avoided in the text.

6) Fig. 5C (legend): p=0.0000.... revised to p<0.0001.

Given the therapeutic potential of the proposed new idea, the authors may want to discuss carefully the hurdles that lie ahead before this application can be used to treat PD patients clinically.

1st Revision - authors' response 29 December 2014

Referee #1 (Comments on Novelty/Model System):

The neuroprotective role of Nurr1 and Foxa2 has not been previously explored, in particular via the action of these transcription factors in glial cells. The main weaknesses of the work is that AAV-mediated gene delivery does not infect glial cells at high efficiency and therefore it is unclear if the in vivo experiment demonstrates neuroprotection via the action of Nurr1 and Foxa2 in glial cells, as suggested by the authors. In addition, the therapeutic efficacy of this approach remains uncertain as the MPTP model does not fully replicate the pathogenesis of Parkinson's disease. Complementing this study with genetic models of PD would be useful, but I understand that this implies a new set of experiments that could be difficult to achieve in a reasonable time frame. It would be for instance interesting to determine if Foxa2 and/or Nurr1 knockdown leads to increased vulnerability to alpha-synuclein. The authors suggest that gene therapy should be envisaged to overexpress these transcription factors. However, some caution is needed as long-term overexpression of transcription factors could lead to deleterious effects that make this approach unlikely to be approved as such. In addition, it is unclear if glial cells actually express Foxa2 and Nurr1 in physiological or diseased conditions, and therefore unexpected long-term effects have to be expected following forced expression in these cell types.

Referee #1 (Remarks):
This article by Oh and colleagues reports on the neuroprotective effects of the combined

expression of Nurr1 and Foxa2 on mouse dopaminergic neurons in vitro and in the MPTP model of Parkinson's disease. They identify an unexpected mode of action of these transcription factors via neighbouring glial cells, and highlight the cooperation between Nurr1 and Foxa2 in neuroprotection. However, it is unclear if Foxa2 and Nurr1 are expressed in glial cells in physiological conditions, possibly in response to oxidative stress for instance, or if the effects observed are only the results of forced expression in these cells. In this case, it would raise some concerns regarding the long-term consequences of transgenic expression of these transcription factors for therapeutic purposes. This is a thorough study, well executed and the manuscript is well written. The following major comments should be addressed before publication:

Authors' response: We are grateful for the reviewer's positive comments. In response to the points raised by the reviewer, we have substantially revised the paper. As a result, our paper is significantly improved. Our point-by-point responses to the reviewer's comments are as follows:

Fig. 1D: the blot showing co-immunoprecipitation of Foxa2 is not convincing. There is no indication of molecular weight in the western blot and the Foxa2 signal is very weak.

Authors' response: We have repeated the immunoprecipitation (IP) analysis, and this time we performed the assays in both directions i.e. IP using anti-Foxa2 antibody followed by WB with anti-Nurr1 antibody, and IP with anti-Nurr1 antibody followed by WB with anti-Foxa2 antibody. Data with a better resolution are shown along with molecular weight indications in the revised Fig. 1D.

Fig. 1F: what are the cells that are TH-negative? Is there evidence for expression of Foxa2 and Nurr1 in non-neuronal cells?

Authors' response:

-The cultures in Fig. 1F were enriched with TuJ1+ neurons, because they were derived by differentiation of neural stem/precursor cells of mouse ventral midbrain early in development (E10.5). Glial cell numbers (GFAP+ and Iba+) were relatively low. Among the neuronal cells, TH+ DA neurons were the most abundant. The other neuronal populations were GABAergic and serotonergic neurons (glutamatergic neurons should have been there, but could not be detected with the available antibodies). The cell composition of the culture is shown in Suppl. Fig. S1E of the revised paper.

-We have carefully examined endogenous Nurr1 and/or Foxa2 expression in glial cells using not only in vitro cultures but also using in vivo adult mouse midbrain sections. We also included experiments to detect factors after toxin-treatment, based on the study of Saijo et al. demonstrating Nurr1 expression induced by LPS in glial cells of the adult midbrain (Cell, 2009). However, we could not detect any GFAP+ astrocytes or Iba+ microglia expressing these factors at all, not even Nurr1, in vitro or in vivo regardless of the presence or absence of toxic stimuli (see the Figures below this reply). It is possible that low levels of Nurr1/Foxa2 proteins are expressed in glia, and that our assays were not sensitive enough to detect them. Another possibility is that endogenous Nurr1/Foxa2 are expressed in glial cells, but were not detected because the glial markers GFAP and Iba-1 had disappeared or were reduced in the Nurr1/Foxa2-expressing glia. It is well known that glial marker expression is frequently altered by changes in the in vivo environment; in particular GFAP expression is reduced (or disappears) during maturation into beneficial astroglial cells (Hirano et al., 1998, J. Neurosci. Res., Liu et al., 2002, Glia, Pringle et al., 2003, Development, Lovatt et al., 2007, J Neurosci., Aglus et al., 2010, Dev. Biol., Loybon et al., 2013, Cell Reports). In agreement with this, Fig. S6 of our study (Suppl. Fig.S5 of the unrevised paper) shows fewer GFAP+ and Iba1+ cells in the midbrain after Nurr1+Foxa2 AAV injection. Thus the endogenous expression of Nurr1 and Foxa2 in glial cells needs to be examined in future studies employing more appropriate/sensitive detection methods, for example, by cell-lineage tracing experiment. Endogenous Nurr1/Foxa2 protein expression in glia is discussed on p20, line 3-10 of the revised text.

Even though endogenous Nurr1/Foxa2 expression in glial cells was not confirmed, this study shows that exogenous expression of these factors in glia has a dramatic neuro-protective effect on degenerating mDA neurons. Thus our study should be judged in relation to its potential therapeutic value. We agree with the reviewer that the non-physiologic expression of Nurr1/Foxa2 could cause side effects, and thus examination of potential long-term adverse effects is required. We consider this issue in the discussion of the revised manuscript (p20, line 3-10).

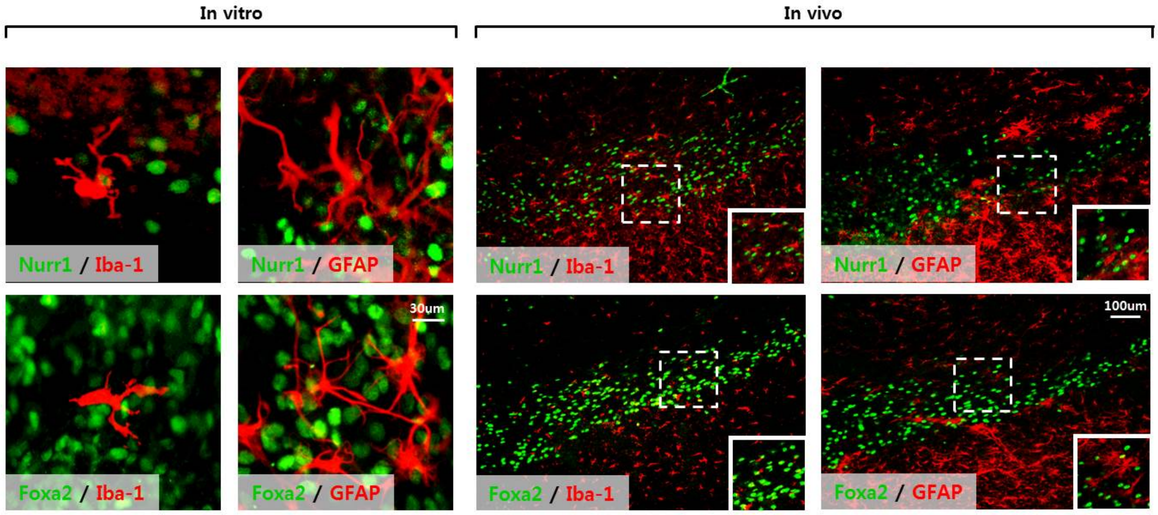

Figure. Endogenous Nurr1 and Foxa2 expressions were not detected in Iba-1+ microglia and GFAP+ astrocytes derived from in vitro cultures (left) and in vivo adult midbrain brain sections (right).

Fig. 1G: the authors should provide evidence for the efficacy of the shRNA against Foxa2 and Nurr1. Knockdown could be assessed by immunocytochemistry with specific antibodies. Also, it is unclear if the number of TH+ cells is decreased in shN, shF and shNF in the absence of any exposure to H2O2.

Authors' response:

- We thank the referee for pointing out the missing data. The effects of the shRNA treatments are now described with specific data on p6, line 14 - p7, line 3 of the revised text.

-TH+ cells were synergistically decreased by shNurr1 and shFoxa2 treatment even in the H2O2-untreated cultures (1235±17 (shcontrol), 816±13 (shNurr1), 805±14 (shFoxa2), 276± 7 cells/well (shNurr1+shFoxa2), n=4 culture wells in each group), indicating that Nurr1 and Foxa2 cooperate in promoting mDA neuron survival. The data on DA neuron numbers are presented on p6, line 23-p7, line 3 of the revised text.

Because TH+ cell numbers in the shControl, shNurr1, shFoxa2, and shNurr1+Foxa2 were different in the absence of toxin, the effects of shRNA on the mDA neurons resisting H2O2-induced toxicity shown in Fig. 1G were estimated from the percentages of the TH+ cells in the respective H2O2-untreated cultures (H2O2= 0 uM).

Most of the in vitro experiments using H2O2 as a stressor find significant effects of changes in Foxa2 and Nurr1 expression at H2O2 doses of 500µM. This is a non-physiological dose of H2O2 that may engage processes that are very different from the in vivo situation. This point should be at least discussed. Fig. 5: as the forced expression of Foxa2/Nurr1 is expected to induce

expression of dopaminergic markers, the number of Nissl-positive neurons should be quantified. This would help to determine what is the contribution of de novo expression of dopaminergic markers in the substantia nigra and determine the exact extent of the protection of resident neuronal cells.

Authors' response:

-We appreciate these comments. We now discuss the problem of our use of non-physiologic levels of H2O2, and the need for further studies in more physiologic systems on p19, line 21 – p20, line 1.

-In response to the reviewer's comment, we performed Nissl and NeuN-staining. Representative images of Nissl- and NeuN-positive neurons are shown along with TH+ cells in supplementary Figure S5. Similar to TH+ cells, the numbers of Nissl- and NeuN-stained neurons are also greater in Nurr1+Foxa2-AAV injected sides than those of the control, confirming the cytoprotective effect of Nurr1+Foxa2.

The authors do not seem to use unbiased stereology for the assessment of the number of neurons in the substantia nigra, although stereology is now considered as the method of choice in the field. It is unclear what is the number of sections counted in this study. This should be better reported in the Material and Methods section.

Authors' response:

- We actually used the principle and formula of stereology in counting the TH immunoreactive cells, although not using a computerized system. To derive TH+ DA neuronal numbers in 3-demensional midbrain tissues from the TH+ cell counts in 2-dimensional midbrain sections, we applied the Abercrombie correction factor [$N = n$ X $T/(T + D)$], where N is the actual number of cells, n is the number of nuclear profiles, T is the section thickness (30μm), and D is the average diameter of nuclei (p23, line 14-18 in the original and p27, line 3-6 in the revised texts). This is the basic concept and formula used in stereologic cell counting to compensate for double counting in adjacent sections.

-We counted TH+ cells in a total of 11-14 sections throughout the midbrain of each animal. This information is now given on p27, line 2-3 of the revised text.

It is important to determine the in vivo effects of the forced long-term expression of Foxa2 and Nurr1 in the substantia nigra in the absence of any MPTP-induced lesion. This would determine the possible side effects of such a gene therapy approach. Indeed, constitutive long-term overexpression of these transcription factors is likely to cause non-physiological perturbations of the dopaminergic function.

Authors' response: As mentioned earlier, we agree that non-physiologic expression of Nurr1/Foxa2 may cause side effects, and that long-term analyses to evaluate such potential adverse effects are required. In response to the reviewer's point, we examined this issue briefly using 5 mice injected with Nurr1+Foxa2-AAVs on their right sides without an MPTP lesion, and observing their daily behaviours for 2 months. We did not notice any abnormal daily behaviours. In addition, apomorphine injection did not cause any abnormal rotation in these mice and TH+ mDA neurons were symmetrically distributed on the two sides of the midbrain 2 month after the AAV injection (see the Figures below this reply). Although we have addressed this issue superficially, the results are clearly insufficient to completely rule out side effects of forced Nurr1+Foxa2 expression, and additional systematic experiments are required. We hope that the reviewer will understand that such an analysis could not be carried out in the time frame of this revision. As stated earlier, we discuss this issue on p20, line 7-10 of the revised manuscript.

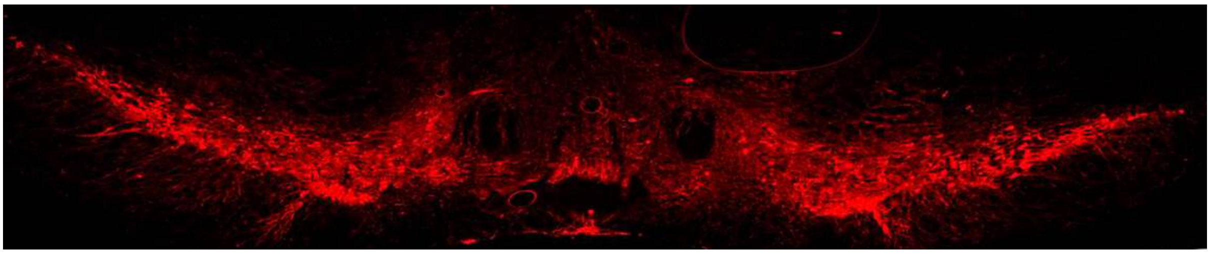

Figure. TH+cell image in the midbrain section of the mouse injected with Nurr1+Foxa2 AAV (right) and control AAV (left) 2months after the virus injection.

Supplementary figure S3: the tropism of the AAV vector used in this study should be better reported, if possible with a quantitation of the neuronal vs glial transduction. It is stated in the discussion that AAV transduces less than 5% of glial cells. It would be useful to determine the percentage of GFP-positive cells that are TH+, GFAP+ and Iba1+. This is important as the authors suggest that some of the observed neuroprotective effects are due to changes in the glial rather than neuronal cells. If less than 5% of the transduced cells are glial cells, it is unlikely that glial cells may play a prominent role in the observed in vivo results.

Authors' response:

-The quantified data are now shown in p19, line2-5 of the revised text.

-Since AAV-mediated transgene expression in glia was not high, we agree that glial cells may not play a prominent role in the observed in vivo results. This issue is discussed along with the need to develop AAV vectors with better glial tropism (p17, line 17-20 of the original text and p18, line 24 – p19, line 7 of the revised text). On the other hand, it is possible that we failed to detect some glial cells expressing the AAV-mediated transgene for the following reasons. First, as mentioned earlier, it is well known that marker expression varies in glial cells and can be affected by changes in the environment (Allaman et al., TINS 2011; Sofroniew, TINS 2009). Thus glial cell detection by immunostaining with specific antibodies may be inaccurate (Emsley and Macklis, Neuron Glia Biol., 2006). Second, a large proportion of AAV-infected GFP+ cells (26 %) were not unidentified in our specific immunostaining experiments (negative for all of TH, GFAP, Iba-1). Thus, it is quite possible that glial cells expressing the exogenous Nurr1+Foxa2 were more numerous than those detected by GFAP and Iba-1 immunostaining, and that the Nurr1+Foxa2 transgenes in the identified plus unidentified glia together generated a significant neuroprotective environment. This is discussed on p19, line 7 - 18 of the revised text.

The genes Foxa1 and Foxa2 have been reported to have redundant functions. In the Foxa2 knockdown experiments, what is the status of Foxa1?

Authors' response: We expected Foxa1 expression to show a compensatory increase in response to shFoxa2 treatment. However, surprisingly, Foxa1 mRNA expression was reduced to about the same extent as Foxa2. We confirmed this on 4 independent sample sets using 2 different PCR primer sets (Foxa1 mRNA levels: 1.0±0.13 in the shcontrol-treated vs 0.37±0.06 in the shFoxa2-treated cultures, n=12 PCR reactions from 4 independent sample batches). We note that a positive regulatory loop between Foxa1 and Foxa2 expression has previously been suggested based on the study of KO mice (Mavromatakis et al., Mech. Dev. 2011).

Minor comments:

Fig. 3B: the description of the statistics on the graph is not very clear.

© EMBO

Authors' response: The statistical analysis is now more clearly described in the revised legend to Fig. 3B (p38, line 14-17).

Fig. 5D: in contrast to what is stated in the Result section, apomorphine is indicated to induce "contralateral" rotations. If this means contralateral to the AAV-injected side, it appears unlikely to be the case.

Authors' response: We used 'ipsilateral' for the direction towards the Nurr1+Foxa2-AAV (NF-AAV)-injected side (right, clockwise). Thus the 'contralateral rotation' shown in Fig. 5D is incorrect, but the 'ipsilateral rotation towards the NF-AAV-injected side' in the text is correct. We are so sorry for the mistake and have corrected it in the text (p14, line 13) and Fig. 5D.

Fig. 5E: please clarify how asymmetry in the cylinder test is expressed.

Authors' response: Mice were placed in a small transparent cylinder (height, 15.5 cm; diameter, 12.7 cm), and the numbers of right and left forelimb contacts with the wall of the arena while rearing were recorded for 3 min (p27, line 13-16). The mice injected with NF-AAV on the right side (C-AAV on the left side) used their left forelimbs more often when rearing against the wall than did the mice injected with the control AAV on both sides (Fig. 5E), while the numbers of right forelimb contacts were not significantly different between the groups (15.5±2.3 vs 16.7±1.9). This is now more clearly described in the revised text (in p14, line 15-20) and Fig. 5E legend (p40, line 19-20).

It is unclear how the vector titre was determined. Did the author use the number of particles containing a genome as determined by real-time PCR? In the group injected with the Foxa2 and Nurr1 vectors, did the authors double the injected dose of AAV vector as compared with the groups injected with individual vectors? This should be more accurately stated in the Material and Methods section.

Authors' response:

-The method for lentiviral titre determination was described in the original manuscript (p22, line 18-21 in the revised text), but that for the AAVs was not. We are sorry for the omission. The method for AAV viral titre determination is now described (p23, line 3-5 of the revised text).

- It is correct that our co-expression experiments involved infecting cells with mixtures of the individual viral preparations (1:1, v:v). Specifically, 1 ul of Nurr1-AAV was mixed with 1 ul of Foxa2-AAV, and the mixture (2 ul) was injected into the midbrain (NF-AAV group). Similarly, 1 ul aliquots of Nurr1-AAV and Foxa2-AAV were mixed with 1 ul of Control-AAV in the Nurr1- and Foxa2-injected groups, respectively, while 2 ul of control-AAV was injected as the control. This experimental design was used to exclude effects (cell toxicity) of differences in viral titre. This is described in the Materials and Methods of the revised text (p26, line 11-14).

What is the promoter used to express Foxa2 and Nurr1 with AAV vectors in vivo?

Authors' response: The CMV promoter was used (p22, line 22-24 of the revised text).

Typographical mistakes:

Abstract section:

Line 9: "protects"
Line 12: "nigrostriatal"

Page 3, line 6: "nigrostriatal"

"transgene" should be used instead of "exogene" throughout the manuscript.

Page 36: "*p=0.0000... " should be corrected

Page 36, line 9: "microscopic"

Authors' response: We have corrected these errors.

Referee #2 (Remarks):

In the following manuscript, Oh and co-workers report the synergistic neuroprotective effect of Nurr1 and FOXA2 in an animal model of PD. Their rational seeds from an observation that Nurr1 and FoxA2 transcription factor expression is lost or decreased, with age in mice; and that knock-down of both TFs dramatically decrease TH expression while increasing cell death, in vitro. Using a battery of in vitro and in vivo tests, they demonstrate that overexpression of both TFs in DA neurons or glia is neuroprotective to DA neurons, and in case of mis-expression is associated with reduced inflammation which is a process otherwise known to exacerbate DA neuron death. Remarkably, the authors show that overexpression of both TFs in vivo prevents DA neuron death in response to MPTP treatment. My only main concern is that all observations made in vitro may results from on-going neurogenesis, rather than protecting TH neurons from death, especially since embryonic cells were used. Time course studies would clarify this issue.

The manuscript otherwise reads wells; the message is simple and straightforward; the research is novel since most experiments were conducted in adult animals, although the authors use embryonic cells and cell lines to confirm or give a rational to some of their work, which in some instances may not be relevant.

Authors' response: We are grateful for the reviewer's positive comments. Our point-by-point responses to the reviewer's comments are as follows:

Comments to authors:

Figure 1: Panel D: Blots really need improvement

Authors' response: We have repeated the immunoprecipitation (IP) analyses, and this time we performed the assays in both directions: IP using anti-Foxa2 antibody followed by WB with anti-Nurr1 antibody, and IP with anti-Nurr1 antibody followed by WB with anti-Foxa2 antibody. Data with better resolution are in the revised Fig. 1D.

Fig. 1, Panel G: - Pictures are misleading: DAPI staining reveals an increase in the number of cells with shNF targeted KO (2-3 fold compare to control). Since the number of C-Capase3 positive cells also increases, the ratio of cell death seems null. Can the author comment on that? - Data should also be presented as % of DAPI, not only TH+ per well (this is not indicative at all).

Authors' response:

-Total DAPI+ cells were decreased by knocking down Nurr1 and Foxa2: DAPI+ cells/well were 19,010, 16,001, 17,737, and 15,972 cells in the shControl (shC), shNurr1 (shN), shFoxa2 (shF), and shNurr1+Foxa2 (shNF)-treated cultures, respectively. We are sorry that the DAPI-stained images of the shC-treated culture did not reflect the real level of DAPI+ cells. We have replaced them with more representative DAPI+/Cleaved Caspase3+ images in the revised Fig. 1G.

-We agree that presenting the % ofDAPI+ cells makes more sense. Thus, the effects of shRNA treatment on cell apoptosis are estimated from the % cleaved caspase 3+ cells among total DAPI+ cells in the revised Fig. 1G.

In contrast, the effects of Nurr1 and/or Foxa2 knock-down on the resistance of DA neurons to H2O2-induced toxic stimuli are estimated from the % TH+ cells in the corresponding H2O2-untreated cultures (H2O2 = 0 uM), because the initial TH+ cell numbers in the shC, shN, shF, and shNF-treated cultures were already different in the absence of H2O2 treatment (1235±17 (shC), 816±13 (shN), 805±14 (shF), 276± 7 cells/well (shNF), as described on p6, line24-p7, line 3 of the revised text).

Fig. 1, Panel G: - The authors need to add images for appreciation of Nurr1 and FOXA2 downregulation following sh KO. Does shNurr1 decrease FOXA2 expression? Does shFOXA2 decrease Nurr1 expression? → Show pictures.

Authors' response: We appreciate the referee pointing out the missing data. The effects of the shRNA treatments on Nurr1 and Foxa2 expression are described with the specific data on p6. line 15-20 of the revised text (*we could not find an appropriate place in Fig. 1 to insert the data for the shRNA treatments on Nurr1 and Foxa2 expression*). In qPCR analyses, the mRNA expression levels of Nurr1 were l±0.03, 0.43±0.01, 0.84±0.03, 0.28±0.004 in the shcontrol, shNurr1, shFoxa2, and shNurr1+shFoxa2, respectively (n= 3 PCR reactions for each group), and those of Foxa2 were 1±0.05, 0.89±0.45±0.06, 0.33±0.02, respectively. Consistent with a positive regulatory loop for Nurr1 and Foxa2 expression (Yi et al., Development 2014), Nurr1 expression was slightly decreased by shFoxa2 treatment (0.84) and Foxa2 expression was slightly decreased by shNurr1 treatment (0.89).

Fig. 1, Panel G:

- Are other neuronal subtypes affected by the treatment, in other words are only TH+ cells dying or could interneurons (GAD65/67) present in the culture die at the same rate, speed?
- The authors used sh vectors leading to partial KO (reduction of 30-40%), moreover, it is possible that double infection with shNurr1 and shFOXA2 viruses is toxic per se. What MOI was used in these experiments? Could the authors show that neuronal death is TH neurons-specific?

Authors' response:

- In our specific experimental conditions, the shNurr1 and shFoxa2 treatments did not significantly affect the survival of GABA and serotoninergic neurons (glutamatergic neurons should also be present but were not detected by immunostaining), probably because Nurr1/Foxa2 are mainly expressed in DA neurons in the differentiated VM-NSC cultures.

- Titres of the lentiviruses were determined using a QuickTiter^{TM} HIV Lentivirus Quantitation Kit (Cell Biolabs, San Diego, CA). Two hundred ul/well (24-well plates) with 10^6 transducing unit (TU)/mL (60-70ng/mL) were used for each transduction reaction (described on p19, line 18-19 of the unrevised and p22, line 17-18 of the revised texts). Coexpression studies were carried out by infecting cells with mixtures of the individual viral preparations (1:1, v:v) (described on p23, line 4-

5 of the revised text). Specifically, 200 ul of shNurr1 was mixed with 200 ul of shFoxa2, and the mixture was added to the shNF-cultures of Fig. 1G. Similarly, 200 ul of shNurr1 or shFoxa2 was mixed with 200 ul of shControl, respectively, and the mixtures were added to the shN- or shF-treated cultures, while 400 ul of shControl was used as control. This experimental design was used to exclude differences in toxicity caused by using different viral titres.

- Have the authors checked that the decrease of TH+ cells is not due to 1) lack of on-going TH neuron genesis due to F and N KO or 2) lack of maturation of the progenitors, which undergo neuronal death, as they cannot mature (were BrdU pulses performed?). It is highly possible, since embryonic cells were used, that the shN, shF and shNF KO prevent maturation of progenitors. Is the number of TH+ cells identical for all conditions on day 1, prior to sh KO?

Authors' response: Because we performed all our experiments on fully differentiated VM-NSC cultures, we did not expect that Nurr1 and Foxa2 effects on DA neurogenesis would be involved in our findings. However, the reviewer is right that this possibility needs to be ruled out. Hence we have performed BrdU pulse experiments during the differentiation of the VM-NSC as the reviewer suggested (the method is described in p21, line10-11 of the revised text). None of TH+ cells were labelled with BrdU on differentiation days 6-9 when the analyses in Fig.1 was performed, pointing to no new DA neuron formation in the cultures. Furthermore, the shNurr1 and shFoxa2 treatments did not induce any TH+/BrdU+ cells. These data are now included in Suppl. Fig.S1 of the revised paper.

Figure 2: Panel A and panel D:

- TH staining intensity for young (panel A) and "minus MPTP" should be identical, since the age of animal is identical.

- Data on decrease in intensity should be completed with TH+ quantification (preferably by stereology).

- FOXA2 intensity panel A and B should be identical since animals are of same age. If those are 2 different batches, data should be presented as % of control.

- Why is TH staining lost at 18 months of age? Dawson and Dawson (nature neuroscience 2013) show sustain staining intensity for TH in animals aged 20 months. Could this be strain specific? Why are Nurr1 and FOXA2 staining brighter in panel D (minus MPTP) compare to panel A 10 weeks young?--> those should be identical.

Authors' response:

-We appreciate these comments. We have replaced the midbrain images for young mice in Fig. 2A by more representative Nurr1/TH and Foxa2/TH images, with immunoreaction intensities similar to those of Fig.1 D without MPTP treatment (-).

-To directly compare Nurr1 and Foxa2 expression levels in the midbrain DA neurons of young and old mice (same strain, BL/6), we performed all the experimental procedures for the young and old animals in parallel. This led to the conclusion that Nurr1 and Foxa2 expression levels in TH+ DA neurons decreased significantly in old mouse midbrains. In addition, as the reviewer judged from the Fig. 2A images, we realized that TH expression also seemed to be substantially reduced in the aged midbrains. However, we did not quantify these, because the focus of our study was on Nurr1/Foxa2 expression in DA neurons, not TH levels. The reviewer points out that the age-dependent reduction of TH expression is not consistent with the data of Dr. Dawson's group (Nature Neursci. 2013) demonstrating identical midbrain DA neuronal numbers in mice at 2-20 months, and therefore suggests that we quantify TH+ DA neuronal numbers to clarify matters. Basically we appreciated the reviewer's suggestion, and tried to do this with the slides already stained for TH/Nurr1 and TH/Foxa2. However, when we examined the stained slides, the TH staining had already faded. Re-staining did not seem possible without destroying the tissues. Because we had used up all the aged

tissue sections, we would have had to wait for almost 1.5 year to obtain completely age-matched mice. We think that the issue of age-dependent changes in midbrain DA neuron numbers and TH expression levels had better be addressed in a separate study.

Fig. 2. Panel B:

- Bands for FOXA2 missing while a decrease is mentioned in figure legend and text; please correct (also, indicate molecular weight of the bands).

Authors' response: We are sorry for the missing Foxa2 WB image. Indeed, it was there and still is there in our original figure file. We were therefore surprised to find that it had disappeared from the submitted version of the figure. We guess that it was lost during the uploading process when the figure file was converted to a format acceptable for submission. We will carefully check the Foxa2 WB image included in the current Fig. 2B.

Fig. 2

- Is TH maintained or also decreased? Please add Western Blot data for TH.

Authors' response: A TH WB image is included in the revised Fig. 2B.

Fig. 2. Panel C and E:

- Add TH quantification

- Are Nurr1 and FOXA2 expression lost because TH neuron age? Counting shows a 2 fold decrease in F and N staining, but images report a complete loss of the markers: what is the proportion of TH still expressing the TFs at D24?

- Are other DA determinant decreased or lost? Lmx1a, lmx1b, En1 (→ this could be checked by RT-PCR or qPCR). How about neuronal markers VMAT2, AADC?

Authors' response:

- The images for Nurr1+, TH+ cells and Fox2+,TH+ cells have been replaced by ones more representative of the quantification data.

- Data for % Nurr1 and Foxa2-expressing cells out of total TH+ cells are described on p8, line 2-3 of the revised text.

-Based on the reviewer's comment, qPCR analyses of other DA neuronal/midbrain markers were carried out at D12 and D24, and the data demonstrating reduced expression of those genes after longer culture times are shown in the graphs below. However, we could not add these results to the main data in Fig. 2, due to space limitations, given that the main focus of the figure is the reduction of Nurr1 and Foxa2 expression after aging and toxic insult.

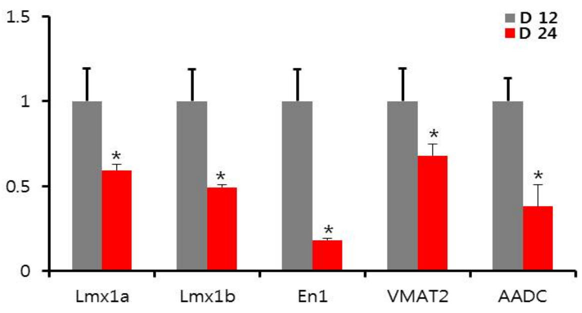

*Figure. Realtime PCR data for the expression of the other dopamine neuron markers at D12 and D24 of the culture. *P<0.05*

Figure 3:

- Panel D: is increase in TH due to on-going neurogenesis? Have the authors tried to block cell proliferation to really prove maturation occurs? Have the authors exposed the cultures to BrdU and stain for BrdU /TH to rule out on-going TH neuron genesis? One way to address neurogenesis is to present TH counts out of DAPI (absolute, per field of view), and as ratio TH/DAPI: if ratio close to one, one can conclude on-going neurogenesis occurs, leading to increase in absolute number of TH+ cells.

Authors' response: As described on p18, line 16-17 of the original text (p21, line 18-19 of the revised), Ara-C was added to eliminate proliferating cells in the primary midbrain DA neuron cultures used for the experiments in Fig. 3. We confirmed that none of TH+ cells was labelled with BrdU even after 48 hr of a BrdU pulse (actually none were BrdU+), confirming the lack of on-going DA neurogenesis in the cultures.

- RT-qPCR for anti-oxidant in cultures treated with MPP+ and 6-OHDA are missing.

Authors' response: The qPCR data are included in Suppl. Fig. S2B.

Figure 4:

- Panel A: As opposed to previous work from Saijo et al (2009, Cell), Nurr1 has a little neuroprotective effect; could the authors comment on this discrepancy?

Authors' response:

-We don't think that Nurr1 had only a small effect in our study. Our data show a substantial neuroprotective effect of forced Nurr1 expression, although it was not as dramatic as that of Nurr1+Foxa2 co-expression: the effect of Nurr1 on proinflammatory cytokine expression in microglia was especially dramatic, as seen in Fig. 4A.

-Saijo et al (Cell, 2009) observed the effects of Nurr1 transiently induced by LPS treatment in glial cells in loss-of-function experiments. By contrast, we examined the therapeutic effect of forced Nurr1 expression. Thus, the extent of the Nurr1 effects seen in the gain-of-function study cannot be directly compared with the effects seen in the loss-of-function study.

Figure 4:

- All panels together show that NF overexpression dramatically increases expression (probably secretion) of neurotrophins, and is associated with decrease expression of pro-inflammatory mediators. This in vitro data were unfortunately not confirmed in vivo. Therefore, the reviewer is still not sure what this figure adds to the study. Could some of the findings presented in this figure be extended to the in vivo part (e.g counting IBA1 staining (reduction should be observed when NF are injected in the SN of the animals), staining for growth factors, etc)?

Authors' response: The in vivo data comparing the expression of the pro-inflammatory mediators and the neurotrophic factors in the NF-AAV-injected and control-AAV-injected brains are shown in Suppl. Fig. S6 of the revised paper (Suppl. Fig. S5 of original unrevised paper). In addition, based on the reviewer's suggestion, we have added images of Iba-1+ cells and the expression data to the supplementary figure.

- To the reviewer's knowledge, SHH is by default increased with inflammation; has this been tested (LPS treated vs non-LPS treated)?

Authors' response: In response to this point, we have performed qPCR for SHH expression in cultures treated with LPS and controls. We did not detect any significant difference in SHH expression between the groups (0.98±0.09 in LPS-untreated vs 1.0±0.08 in LPS-treated, n=3 PCR reactions).

Figure 5:

- Overall comment:

**** Stereological counting is mandatory when working with animal sections (panel B: TH quantification).***

Authors' response: - We actually used the principle and formula of stereology in counting the TH immunoreactive cells, although not using a computerized system. To derive TH+ DA neuronal numbers in 3-demensional midbrain tissues from the TH+ cell counts in 2-dimensional midbrain sections, we applied the Abercrombie correction factor [$N = n \text{ X } T/(T + D)$], where N is the actual number of cells, n is the number of nuclear profiles, T is the section thickness (30μm), and D is the average diameter of nuclei (p23, line 14-18 in the original and p27, line 3-6 in the revised texts). This is the basic concept and formula used in stereologic cell counting to compensate for double counting in adjacent sections.

Figure 5:

**** High magnification images are needed to appreciate the number of TH+ neurons and their morphology.***

Authors' response: We appreciate this point and have added high-power TH+ cell images as insets.

**** Why was not the effect of each single factor assessed? Perhaps, and in light with the work conducted by Decressac and co-workers, either Nurr1 or FOXA2 would have been sufficient to induce cellular and locomotor recoveries.***

Authors' response: The neuroprotective effects of single Nurr1-AAV and Foxa2-AAV injections were estimated and are shown in Suppl. Fig. S4 of the unrevised and revised paper.

**** Assessment of inflammation is required to give a rational to data presented in figure 4: GFAP, IBA1 quantifications are necessary here.***

Authors' response: As stated above, in vivo data comparing the expression of pro-inflammatory mediators including GFAP and IBA1 are shown in Suppl. Fig. S5 (now Suppl. Fig. S6 in the revised paper).

Figure 5:

- Panel M: what cells are targeted with the AAV employed? Are they mainly neurons or glia? Is the effect of combined overexpression of N and F direct or indirect? If AAV used, the injections should be performed in the striatum to have a specific expression of both TFs in DA neurons.

Authors' response: AAV-mediated transgene expression was preferentially detected in (mDA) neurons, while the proportion of glial cells expressing the transgene was not high. The neurotropism of AAVs is well known (reviewed in (McCown et al., Br Res., 1996). The development of vector systems with better glial tropism is required to improve the ability of N+F to polarize glial cells towards the M2 type, which would create a neuroprotective environment surrounding degenerating mDA neurons. These issues are discussed on p17, line 17-23 of the unrevised and p18, line 24 - p19, line 18 of the revised text.

- Panel P: assessing TH expression 14 days post-treatment and lesion is rather quick. Why not assessing TH counts at 4 and 8 weeks as well (like in panel A)?

Authors' response: In response we have performed another set of the experiments measuring TH+ cells 4 and 8 weeks after MPTP treatment (see revised Fig. 5O&P).

Referee #3 (Comments on Novelty/Model System):

the authors show an impressive amount of in vitro and in vivo data to support that elevating the expression of Nurr1 and Foxa2 has a synergistic effect not only in improving PD-related symptoms but also in improving mDA neuron survival. Overall, this is a well-designed and nicely performed study with a focus of high biological and translational interest.

Referee #3 (Remarks):

This work by Oh et al reports a new way, i.e. co-expression of Nurr1+Foxa2, to improve the survival of mDA neurons in PD models. As opposed to current PD therapies, this approach has the potential of changing the disease course in addition to symptomatic improvement. In this manuscript, the authors show an impressive amount of in vitro and in vivo data to support that elevating the expression of Nurr1 and Foxa2 has a synergistic effect not only in improving PD-related symptoms but also in improving mDA neuron survival. Mostly importantly, the neuroprotective effect of Nurr1+Foxa2 is sustained after 6 months. In addition, this study also elucidates the cellular and molecular mechanisms that underlie the effect of Nurr1+Foxa2 co-expression. It shows that Nurr1+Foxa2 acts by suppressing the secretion of pro-inflammatory cytokines and increasing the secretion of neurotrophic factors in neighbouring glia and by up-regulating Nrf2 and anti-oxidant genes in mDA neurons. The authors present their findings logically and clearly. Overall, this is a well-designed and nicely performed study with a focus of high biological and translational interest. I only have a few minor suggestions that need to be addressed.

Authors' response: We greatly appreciate for the complimentary comments. In addition, we thank for suggesting the appropriate and helpful points. Based on the points raised by the reviewer, we substantially revised the paper. As a result, our paper is significantly improved. Our point-by-point responses for the reviewer's comments are as follows:

1) It would be nice to add a simple diagram in the end that summarizes the cell-autonomous and paracrine activities of Nurr1+Foxa2 on VM neuron and glia.

Authors' response: In response we have generated a schematic drawing summarizing the Nurr1+Foxa2 effects seen in this study (Fig. 6).

2) Double check the registration of the boxed areas with the enlarged panels in Fig. 1E, Fig. 1G, and Fig. 2A.

Authors' response: We were sorry to find that the boxed areas and the insets in the indicated images were not completely identical. We have now fixed this.

3) In Fig. 1G, it is better to provide color-coded names of the gene products (TH, Casp3, DAPI) directly within the images.

Authors' response: The color-coded names have been added.

4) The protein signal for Foxa2 is missing in Fig. 2B western blot.

Authors' response: We are sorry for the missing Foxa2 WB image. Indeed, it was there and still is there in our original figure file. We were therefore surprised to find that it had disappeared from the submitted version of the figure. We guess that it was lost during the uploading process when the figure file was converted to a format acceptable for submission. We will carefully check the Foxa2 WB image included in the current Fig. 2B!

5) Single letter abbreviations, such as N and F, should be avoided in the text.

Authors' response: We have changed the abbreviations to the full names throughout the text.

6) Fig. 5C (legend): p=0.0000.... revised to p<0.0001.

Authors' response: We corrected it in the revised Fig.5 legend (p40, line 14).

Given the therapeutic potential of the proposed new idea, the authors may want to discuss carefully the hurdles that lie ahead before this application can be used to treat PD patients clinically.

Authors' response We are grateful for this comment. We now consider these hurdles at the end of the discussion section (p19, line 20 – p20, line 10 of the revised text).

2nd Editorial Decision 26 January 2015

Thank you for the submission of your revised manuscript to EMBO Molecular Medicine. We have now received the enclosed reports from the referees that were asked to re-assess it. As you will see the reviewers are now globally supportive and I am pleased to inform you that we will be able to accept your manuscript pending the following final amendments:

Please carefully address the last set of comments from Referee #1.

Please submit your revised manuscript within two weeks. I look forward to seeing a revised form of your manuscript as soon as possible.

***** Reviewer's comments *****

Referee #1 (Remarks):

Review of Oh et al, EMBO Mol Med 2015
Comments to authors

The authors have adequately answered most of my questions. Here are my remaining comments:

Suppl. Fig. 1D
The VMAT2 staining appears to label all cells. This is surprising as it is expected to colocalize with TH. I would suggest to remove this panel as this result could be due to false positive staining.

Page 20, line 7: please correct "data now shown"

My main concern is that the counting of neurons was not performed using computerized stereology. Although the Abercrombie formula corrects over-counting due to cell sectioning, it is a biased formula, which does not replace unbiased stereological counting of neurons, the main method currently accepted in the field. The same remark applies to both counts of TH+ cells as well as evaluation of the number of Nissl-positive neuronal nuclei in the substantia nigra.

Referee #2 (Remarks):

The authors have addressed all reviews, which led to major improvement of the manuscript.
In its current shape, I consider the manuscript suitable for publication

2nd Revision - authors' response 10 February 2015

Referee #1 (Remarks):

Comments to authors

The authors have adequately answered most of my questions. Here are my remaining comments:

Authors' response: We are grateful for the reviewer's positive comments.

© EMBO

Suppl. Fig. 1D
The VMAT2 staining appears to label all cells. This is surprising as it is expected to colocalize with TH. I would suggest to remove this panel as this result could be due to false positive staining.

Authors' response: Based on the reviewer's suggestion, the VMAT2-staining image was removed in Suppl. Fig. 1D.

Page 20, line 7: please correct "data now shown"

Authors' response: Thank you for pointing out the typographical error. We were informed from the editor that "data not shown" comment is not permitted in "EMBO Molecular Medicine". Thus we removed it.

My main concern is that the counting of neurons was not performed using computerized stereology. Although the Abercrombie formula corrects over-counting due to cell sectioning, it is a biased formula, which does not replace unbiased stereological counting of neurons, the main method currently accepted in the field. The same remark applies to both counts of TH+ cells as well as evaluation of the number of Nissl-positive neuronal nuclei in the substantia nigra.

Authors' response: Considering that stereological counting is based on Abercrombie formula, we believe that cell counting with Abercrombie correction can replace computerized stereological counting, and we haven't met any serious criticisms on this way of in vivo cell counting in the reviews for our previous publications. As the reviewer pointed, it is definitely true that computerized stereological counting is the most appropriate unbiased system for in vivo cell counting. However, unfortunately the system is not currently available for us, and thus we are afraid to say that it is not possible for us to address this reviewer's point in our current situations.

Referee #2 (Remarks):

The authors have addressed all reviews, which led to major improvement of the manuscript. In its current shape, I consider the manuscript suitable for publication.

Authors' response: We are very grateful for recommending publication of our paper.
